# Coordinate regulation of the expression of SdsR toxin and its downstream *pphA* gene by RyeA antitoxin in *Escherichia coli*

**DOI:** 10.1038/s41598-019-45998-z

**Published:** 2019-07-03

**Authors:** Jee Soo Choi, Hongmarn Park, Wonkyong Kim, Younghoon Lee

**Affiliations:** Department of Chemistry, KAIST, Daejeon, 34141 Korea

**Keywords:** Bacterial toxins, Small RNAs, RNA, Bacterial transcription

## Abstract

In *Escherichia coli*, SdsR and RyeA, a unique pair of mutually *cis*-encoded small RNAs (sRNAs), act as toxin and antitoxin, respectively. SdsR and RyeA expression are reciprocally regulated; however, how each regulates the synthesis of the other remains unclear. Here, we characterized the biosynthesis of the two sRNAs during growth and investigated their coordinate regulation using *sdsR* and *ryeA* promoter mutant strains. We found that RyeA transcription occurred even upon entry of cells into the stationary phase, but its apparent expression was restricted to exponentially growing cells because of its degradation by SdsR. Likewise, the appearance of SdsR was delayed owing to its RyeA-mediated degradation. We also found that the *sdsR* promoter was primarily responsible for transcription of the downstream *pphA* gene encoding a phosphatase and that *pphA* mRNA was synthesized by transcriptional read-through over the *sdsR* terminator. Transcription from the σ^70^-dependent *ryeA* promoter inhibited transcription from the σ^S^-dependent *sdsR* promoter through transcriptional interference. This transcriptional inhibition also downregulated *pphA* expression, but RyeA itself did not downregulate *pphA* expression.

## Introduction

There are over 100 noncoding small RNAs (sRNAs) in *Escherichia coli*^1–7^; these can be divided into two categories: *cis*-acting and *trans*-acting. *Cis*-acting sRNAs, which are *cis*-encoded, act as antisense RNAs because they bind to sense mRNAs transcribed on the opposite DNA strand, thereby up- or downregulating their expression^8–10^. *Trans*-acting sRNAs have been widely studied and shown to regulate target mRNAs by base pairing with them through seed regions, usually with the help of the RNA-binding protein Hfq^11–13^. A single *trans*-acting sRNA can affect a variety of physiological events by interacting with multiple target mRNAs^6,11,14–17^. Therefore, sRNAs play a pivotal role in coordinating various aspects of cellular metabolism by fine-tuning the expression of their target genes.

Two sRNAs, SdsR and RyeA, have a number of characteristics that make them unique. They are *cis*-encoded sRNAs for each other^4,18–20^, while SdsR also function as a *trans*-acting sRNA^18,21^. SdsR acts as a regulator of multiple mRNAs by base-pairing with *tolC*, *mutS*, and *yhcB* in *E*. *coli*^18,22,23^, and with several mRNAs, including *ompD*, in *Salmonella*^21,24^. Unlike SdsR, RyeA has not yet been reported to function as a *trans*-acting sRNA. Recently, our group showed that SdsR and RyeA act as toxin and antitoxin, respectively^18^. The toxin function of SdsR is mediated by repression of *yhcB* encoding an inner membrane protein, which is involved in cell envelope biogenesis and cell shape maintenance^25–27^. Since both the toxin and antitoxin are sRNAs, the SdsR/RyeA pair represents a novel type of toxin-antitoxin system.

SdsR and RyeA show reciprocal expression patterns^18^. RyeA expression is dominant in the mid-exponential phase, whereas SdsR expression becomes higher starting from the late exponential phase to the stationary phase^4^. Therefore, SdsR, as an RpoS-dependent sRNA, is highly expressed during the stationary phase, but is barely expressed in the exponential phase.

The *pphA* gene, downstream of *sdsR*, encodes a phosphatase, also called PrpA, that is similar to *Salmonella* PrpA^28^ and λ-PP, a phosphoprotein phosphatase of bacteriophage lambda^29^. PphA plays a role in the protein misfolding response pathway by positively modulating the CpxR/CpxA two-component system, which activates *htrA* transcription^30^. It is likely that there are other target proteins of PphA because additional phosphoproteins accumulate in a *pphA* mutant^30^. It has been reported that *pphA* transcription is induced by heat shock and that its promoter, which is located far upstream of the *sdsR* promoter^30^, has some homology to the promoter consensus sequences of RNA polymerase σ^32^-holoenzyme (Eσ^32^). However, a subsequent study suggested that *pphA* may not be heat-shock inducible^31^. Expression of *pphA* is also induced during biofilm formation and upon urea stress^32,33^. Therefore, it remains unclear how *pphA* expression is regulated.

In this study, we examined biosynthesis of the two sRNAs during growth and coordinate regulation of SdsR toxin and its downstream *pphA* gene by RyeA. We found that both SdsR and *pphA* expression are under control of the *sdsR* promoter and tightly down-regulated during exponential growth by expression of *ryeA*.

## Results

### Biosynthesis of SdsR and RyeA

**Figure 1:**
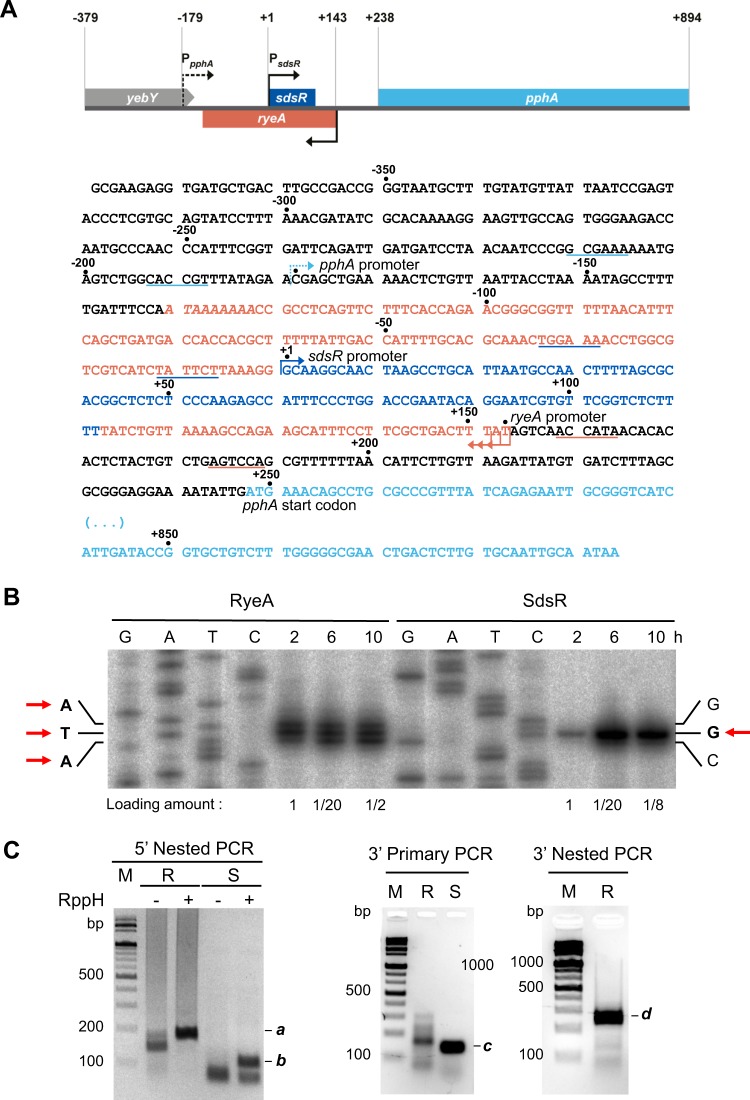
Identification of 5′ and 3′ ends of RyeA and SdsR. (**A**) Schematic representation of the region containing the *sdsR/ryeA* locus and its nucleotide sequence. Promoter regions (−35 and −10) of *sdsR* and *ryeA* are underlined, and their transcription start sites are indicated by arrows. (**B**) Primer extension analysis of *ryeA*-CAT and *sdsR*-CAT fusion transcripts. The regions +341 to +132 (containing the *ryeA* promoter) and −79 to +11 (containing the *sdsR* promoter) were cloned into pKK232-8 to generate *ryeA*-CAT and *sdsR*-CAT plasmids, respectively. Total cellular RNA extracts were prepared from either MG1655 cells containing the *ryeA*-CAT or *sdsR*-CAT plasmid grown for 2 h, 6 h, or 10 h at 37 °C. The ^32^P-labeled primer CAT_R was used to analyze *ryeA*-CAT and *sdsR*-CAT fusion transcripts. Primer extension products were analyzed on a 5% polyacrylamide sequencing gel containing 8 M urea. The DNA ladders (G, A, T and C) were prepared by dideoxy sequencing using the template plasmid DNA and the same primer. Loading amounts are indicated below the lanes. The transcription start nucleotides are indicated by arrows. (**C**) RACE analysis. The 5′ or 3′ RACE products (primary PCR products or nested PCR products) were analyzed on 2% agarose gels. Predicted RACE products were indicated by ***a***, ***b***, ***c***, and ***d***. For 5′ RACE, *E*. *coli* RNA pyrophosphatase (RppH)-treated RNA and untreated RNA were used. M, 100 bp size markers; R, RyeA; S, SdsR.

As there has been some confusion in the literature regarding biosynthesis of SdsR and RyeA in *E*. *coli*^4,15,16,19^, we set to clarify their biosynthesis. To determine the precise transcription initiation sites from each promoter, we subcloned the *sdsR* (−79 to +11 relative to its transcription start site) and *ryeA* (−80 to +10 relative to its most downstream transcription start site) promoter-containing DNA fragments to plasmid pKK232-8 to generate *ryeA*-CAT and *sdsR*-CAT transcriptional fusions, respectively. We then analyzed *sdsR*-CAT and *ryeA*-CAT fusion transcripts using primer extension (Fig. 1A,B). An analysis of primer extension products revealed that the *sdsR* promoter starts transcription at a site 1 nt downstream of the previously reported 5′ end of *E*. *coli* SdsR, which corresponds to the 5′ end of *Salmonella* SdsR^34^. On the other hand, the *ryeA* promoter starts transcription at three sites, of which the most downstream site is the previously predicted 5′ end. In parallel, we performed 5′ RACE experiments using total cellular RNAs with or without the *E*. *coli* RNA pyrophosphatase (RppH) treatment and the RACE products were analyzed on an agarose gel (Fig. 1C). The predicted RyeA band (***a***) was detected at comparable amounts in both RppH-treated and untreated RNAs, whereas the predicted SdsR band (***b***) was observed only in RppH-treated RNA. These results suggest that RyeA and SdsR have different phosphorylation status at the 5′ end: SdsR retains 5′ triphosphate, but RyeA carries 5′ monophosphate. Each RACE band (the corresponding gel area of band ***b*** was used for SdsR in RppH-untreated RNA) was eluted from the gel and subjected to DNA sequencing analysis (Table 1). The 5′ end of SdsR, observed with RppH-treated RNA, not RppH-untreated, corresponds to its transcription start. On the other hand, RyeA showed only one 5′ end corresponding to its most downstream transcription start site regardless of being treated with RppH. These results suggest that while SdsR has a triphosphate at the 5′ end as a primary transcript, RyeA largely exists as a single processed transcript with a monophosphate at the 5′ end, which could be formed by removing 1 or 2 nucleotides, or pyrophophate from three different primary RyeA transcripts.

**Table 1:** Determination of 5′ and 3′ ends of SdsR and RyeA.

		SdsR^a^	RyeA^a^
5′ ends of RNA^b^	**−**RppH^c^	+3 (1), +31 (2), +32 (1), +34 (1), ND^d^ (5)	+1 (11), +33 (1), +36 (3), ND^d^ (5)
	**+**RppH^c^	+1(6), +3(1), +31(1), ND^d^ (2)	+1 (17), +25 (1), +36 (1), ND^d^ (1)
3′ ends of RNA^b^		+100 (3), +101 (10), +102 (2), ND^d^ (5)	+261 (1), +263 (7), +269 (3), +270 (9)

^a^The RACE products a and b shown in Fig. 1C and c and d in Fig. 1D were cloned by T-blunt vector and analyzed by DNA sequencing. In case of SdsR in RppH-untreated RNA, the corresponding gel area of band b was used for analysis.

^b^The positions of both ends at which each RNA terminates are represented relative to the transcription start for SdsR and the most downstream transcription start for RyeA. The numbers in parentheses indicate the frequency of occurrence.

^c^Untreated and treated with RppH are indicated by − RppH and + RppH, respectively.

^d^ND, non-detectable.

The 3′ ends of SdsR and RyeA were analyzed by 3′ RACE (Fig. 1C and Table 1). A sequence analysis of RACE products suggested that SdsR has heterogeneous 3′ ends that terminate at base positions ranging from +100 to +102 relative to its own transcription start site, whereas RyeA has 3′ ends of +261 to +270 relative to the most downstream transcription start site.

**Figure 2:**
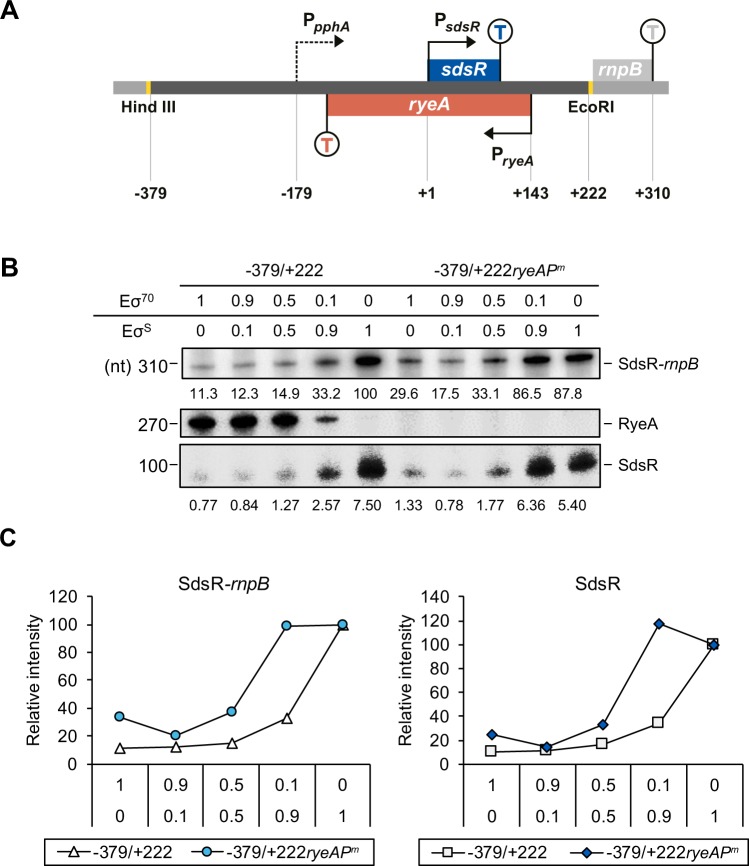
*In vitro* transcription of the *sdsR/ryeA* locus. (**A**) Schematic representation of DNA template used for *in vitro* transcription. The *sdsR* region of −379 to +222 in the *sdsR* promoter-*rnpB* terminator fusion plasmids was shown. Termination sites are indicated by T. (**B**) *In vitro* transcription was performed using different ratio of Eσ^70^ and Eσ^S^ on the *sdsR*(−379/+222) and *sdsR*(−379/+222*ryeAP*^*m*^)-carrying plasmids. The DNA template with the *ryeA* promoter mutation is shown as −379/+222*ryeAP*^*m*^. *In vitro* transcribed RNAs were subjected to Northern blot analysis. The membrane was probed with an anti-RyeA probe for RyeA signals. Then the membrane was briefly washed and reprobed with an anti-SdsR probe without stripping. The asterisks indicate the remaining RyeA signals in the anti-SdsR probed membrane. (**C**) Northern band signals were presented in a line graph along the x-axis with increasing the ratio of Eσ^S^ to Eσ^70^. Relative band intensities of each RNA species are expressed as a ratio to the intensities of the corresponding RNA species transcribed solely by Eσ^S^.

*In vitro* transcription was carried out to determine whether the 3′ ends of SdsR and RyeA correspond to their transcription termini. Plasmid DNA, pSdsR(−379/+222), containing an *sdsR/ryeA* transcription unit consisting of −379 to +222 relative to the *sdsR* transcription start site was used as a template for *in vitro* transcription assays (Fig. 2A). Since the *sdsR* promoter is known to be σ^S^-dependent^24^, we used both RNA polymerase σ^S^-holoenzyme (Eσ^S^) and Eσ^70^. As shown in the left half gel of Fig. 2B,C, Eσ^70^ in the absence of Eσ^S^ (Eσ^70^/Eσ^S^, 1/0) generated almost exclusively RyeA transcripts although it transcribed *sdsR* at about 10% levels as compared to the RyeA transcripts. On the other hand, Eσ^S^ alone (Eσ^70^/Eσ^S^, 0/1) produced only SdsR transcripts without generating RyeA transcripts. These results suggest that transcription of *sdsR* is mostly mediated by Eσ^S^, but that *ryeA* transcription are mediated solely by Eσ^70^. SdsR transcripts of ~100 nt and RyeA transcripts of ~270 nt were detected, indicating that SdsR and RyeA observed *in vivo* are primary transcripts and their 3′ termini are generated by Rho-independent termination. We also found a read-through transcript of ~310 nt that passes over the *sdsR* terminator and terminates at the following *rnpB* terminator^35^, suggesting that genes downstream of *sdsR* could be regulated by *sdsR* transcription.

### Regulation of SdsR and RyeA biosynthesis

**Figure 3:**
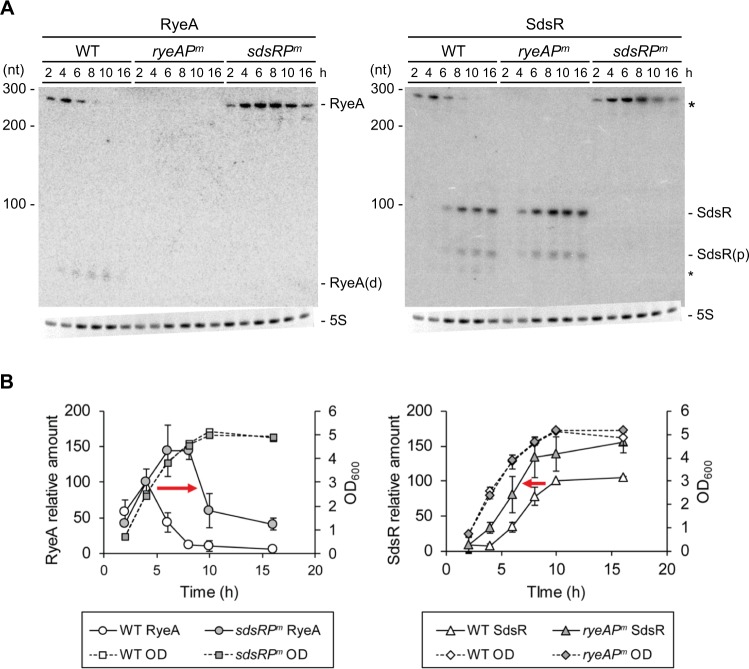
Reciprocal regulation of RyeA and SdsR. (**A**) Effects of the *sdsR* promoter mutation on RyeA and vice versa. Analysis of cellular levels of SdsR and RyeA during growth. Overnight cultures were diluted 1:100 in LB medium and grown at 37 °C. Aliquots of cells were sampled from the cultures at specific time intervals and total cellular RNAs were isolated. Cellular levels of SdsR and RyeA were analyzed by Northern blotting. The membrane was probed with an anti-RyeA probe for RyeA signals. Then the membrane was briefly washed and reprobed with an anti-SdsR probe wiout stripping. SdsR(p), the processed form of SdsR. RyeA(d), a degradation product of RyeA. 5 S, 5 S rRNA. The asterisks indicate the remaining RyeA signals in the anti-SdsR probed membrane. (**B**) Relative band intensities of each RNA are shown with growth times. Relative amounts of RyeA or SdsR are represented as arbitrary units after normalization of the corresponding Northern signals to 5 S rRNA. Cell growth was monitored by measuring optical density (OD) at 600 nm. The change of RyeA expression by the *sdsR* promoter mutation or SdsR expression by the *ryeA* promoter mutation is indicated by an arrow. WT, MG1655 cells; *sdsRP*^*m*^, *sdsR* −10 promoter mutant cells; *ryeAP*^*m*^, *ryeA* −10 promoter mutant cells (mean ± SD; n = 3).

To determine the effects of SdsR on the biosynthesis of RyeA, or vice versa, we constructed *sdsR* and *ryeA* promoter mutant strains in which their −10 elements were inactivated by changing each of them to CTCGAG. We then examined levels of SdsR and RyeA in the promoter mutant cells during growth (Figs 3 and S1). As expected, SdsR and RyeA were not detected in the *sdsR* and *ryeA* promoter mutant strains, respectively (Fig. 3A). In wild-type cells, RyeA was expressed during the early exponential phase, and its expression sharply decreased in the mid-exponential phase. The *sdsR* promoter mutation increased RyeA synthesis during the exponential phase and allowed its continued synthesis in the stationary phase. On the other hand, SdsR expression started in the late-exponential phase in wild-type cells, but appeared at an earlier stage in *ryeA* promoter mutant cells (Fig. 3B). Since the larger *ryeA* covers the entire sdsR, the whole 104-nt SdsR sequence can base-pair with RyeA. We performed analysis of mutual degradation to determine whether interaction between two sRNAs leads to degradation of each other (Fig. S2). We ectopically co-expressed both SdsR and RyeA, but expression of SdsR was increased at a fixed expression level of RyeA, or vice versa. We found that when one is overexpressed, degradation of the other is facilitated, suggesting that this extensive base-pairing leads to degradation of both sRNAs. Because SdsR and RyeA are degraded each other, it is likely that the early appearance of SdsR in *ryeA* promoter mutant cells and the prolongation of RyeA expression to the stationary phase in *sdsR* promoter mutant cells are attributable to the absence of mutual degradation of the two sRNAs. The lack of RyeA degradation products of about 60 nt in *sdsR* promoter mutant cells further supports reciprocal regulation of the two sRNAs through degradation of each other. This mutual degradation could serve to further restrict RyeA expression to the exponential phase and SdsR expression to the stationary phase.

The tight growth phase-specific control of SdsR and RyeA may be needed for SdsR- or RyeA-mediated regulation of certain genes in specific growth phases. To identify such genes, we performed an RNA-seq analysis of *ryeA* and *sdsR* promoter mutant cells as well as wild-type cells. For this RNA-seq analysis, cultures were sampled in both the exponential phase (3 h) and stationary phase (8 h), and genes in *sdsR* or *ryeA* promoter mutant cells that showed a greater than 2-fold change and a p-value < 0.05 were selected for further characterization. We identified 17 genes (7 upregulated and 10 downregulated) in the exponential phase and 5 genes (2 upregulated and 3 downregulated) in the stationary phase for *ryeA* promoter mutant cells (Tables S1 and S2). In contrast, *sdsR* promoter mutant cells showed many changed genes: 171 genes (110 upregulated and 51 downregulated) in the exponential phase and 55 genes (33 upregulated and 22 downregulated) in the stationary phase (Tables S3–5). Selected genes were confirmed by qRT-PCR analysis (Table S6), which showed that RNA-seq data were reliable. A Gene Ontology (GO) analysis showed that many of the genes that were altered by the *sdsR* promoter mutation, either downregulated or upregulated, were related to transport function and encoded membrane proteins (Fig. S3), an observation consistent with previous RNA-seq analysis of SdsR-overexpressing cells^18^.

**Figure 4:**
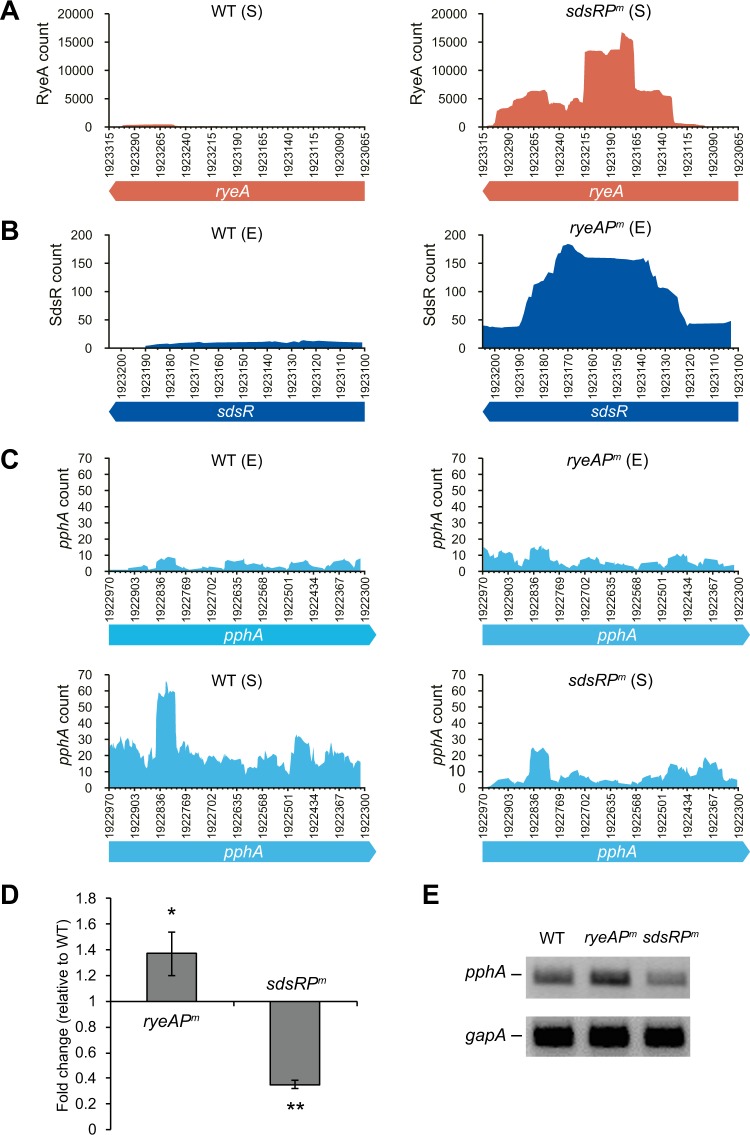
Analysis of RyeA, SdsR, and *pphA* RNA expression. Expression dynamics of SdsR and *pphA* mRNA in *ryeA* promoter mutant cells. (**A**,**B**) Read counts for RyeA and SdsR from RNA-seq data are plotted against *ryeA* and *sdsR* chromosomal positons. WT, MG1655 cells; *ryeAP*^*m*^, *ryeA* promoter mutant cells; *sdsRP*^*m*^, *sdsR* −10 promoter mutant cells. E, exponential phase cells (3 h post-inoculation); S, stationary phase cells (8 h post-inoculation). (**C**) RNA read counts for *pphA* mRNA are plotted against *pphA* chromosomal positons. Since a specific region of higher RNA-seq reads is AU-rich, the higher efficiency of random reverse transcription priming on the AU-rich sequence may cause that transcript depth. (**D**) qRT-PCR analysis of changes in *pphA* mRNA levels by the *ryeA* or *sdsR* −10 promoter mutation. Total cellular RNAs were isolated from WT and the promoter mutant cells at 8 h post-inoculation, and subjected to qRT-PCR. Fold changes relative to WT cells are shown. (**E**) The same total RNAs were subjected to RT-PCR. RT-PCR products were analyzed in a 2% agarose gel. In (**D**), mean ± SD; n = 5–6; *P ≤ 0.05, **P ≤ 0.001 by Student’s t-test.

As expected, the RNA-seq data revealed upregulation of RyeA and SdsR in *sdsR* and *ryeA* promoter mutant cells, respectively (Fig. 4A,B). We also examined the RNA-seq data to see how expression of known SdsR target genes is affected by the *sdsR* or *ryeA* promoter mutation (Table S7). The *sdsR* promoter mutation increased *tolC* and *yhcB* expression by about 2-fold in the stationary phase and exponential phase, respectively. In contrast, the *ryeA* promoter mutation led to a slight decrease in *tolC* and *yhcB* expression, suggesting that RyeA can modulate SdsR-mediated gene regulation. However, *mutS*, another target for SdsR^18,22,23^, did not show gene expression profiles that would be expected from *sdsR* or *ryeA* promoter mutant cells, implying that it may not be a primary target of SdsR.

Interestingly, the RNA-seq data showed that the *pphA* gene, downstream of *sdsR*, was downregulated in *sdsR* promoter mutant cells and upregulated in *ryeA* promoter mutant cells (Fig. 4C). An analysis of *pphA* expression by quantitative reverse transcription-polymerase chain reaction (qRT-PCR) and semi-quantitative RT-PCR confirmed the same downregulation in *sdsR* promoter mutant cells and upregulation in *ryeA* promoter mutant cells (Fig. 4D,E). These data indicate that *pphA* expression may be regulated by the *sdsR/ryeA* regulatory circuit.

### pphA is co-transcribed with SdsR from the sdsR promoter

**Figure 5:**
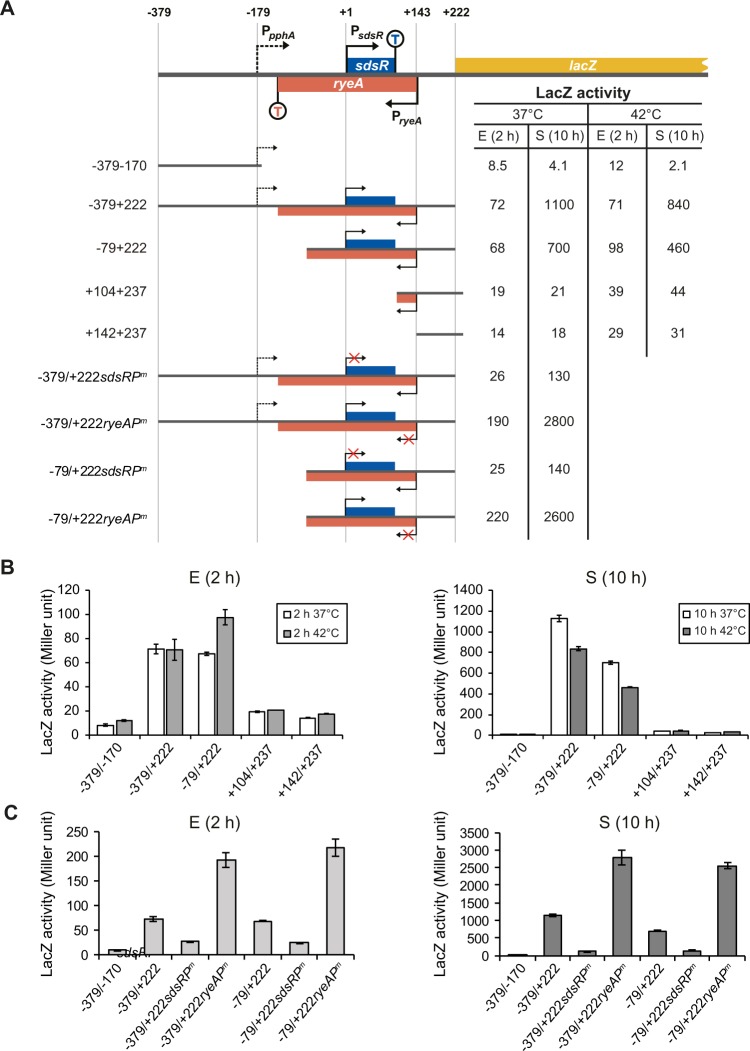
LacZ analysis for *sdsR* and *pphA* transcription. (**A**) Schematic diagrams of various *sdsR*-*lacZ* transcriptional fusion constructs are shown along with their LacZ activities for DJ480Δ*ryeA/sdsR* cells grown for 2 h (**E**) and 10 h (S) post-inoculation at 37 °C or 42 °C. *sdsRP*^*m*^, *sdsR* −10 promoter mutation; *ryeAP*^*m*^, *ryeA* −10 promoter mutation. (**B**,**C**) The LacZ activities are presented in bar graph (mean ± SD; n = 3).

The previously reported *pphA* promoter, located about 180 bp upstream of the *sdsR* promotor, is known as a heat-shock promoter^30^. Therefore, transcription from this promoter should pass through the *sdsR/reyA* locus to transcribe the *pphA* gene; furthermore, the *sdsR* promoter can generate *pphA* transcripts. To determine which promoter makes the greater contribution to *pphA* expression, we constructed various transcriptional *sdsR*-*lacZ* fusions and analyzed their transcriptional activities using LacZ assays (Fig. 5). The promoter region containing a DNA fragment 200 bp upstream and 100 bp downstream (−379 to −170 relative to the 5′ end of SdsR) from the previously known *pphA* transcription site showed little LacZ activity at 37 °C and even at 42 °C. However, a promoter region extended to include the *sdsR/ryeA* locus (+222) exhibited high LacZ activity at 37 °C that was not significantly further increased at 42 °C. Therefore, it is unlikely that the previously reported heat inducible *pphA* promoter is responsible for the observed LacZ activity of the −379/+222 construct. Furthermore, the *sdsR* promoter mutation sharply decreased the LacZ activity of *sdsR*(−379+222)-*lacZ*, indicating that the *sdsR* promoter is a major contributor to *pphA* transcription. The −329 to +222 promoter region was fused to the CAT gene in plasmid pKK232-8 and the 5′ ends of the fused mRNA, transcribed *in vivo*, were analyzed by primer extension analysis (Fig. S4). Most extension products were from *sdsR* transcripts, and no extension products from the reported *pphA* promoter were detected. These data, taken together with *in vitro* data showing read-through transcripts traversing the *sdsR* terminator (Fig. 2B), suggest that the *sdsR* promoter rather than the previously reported *pphA* promoter is responsible for *pphA* transcription.

### Effects of ryeA transcription on expression of SdsR and sdsR-pphA dicistronic mRNA

**Figure 6:**
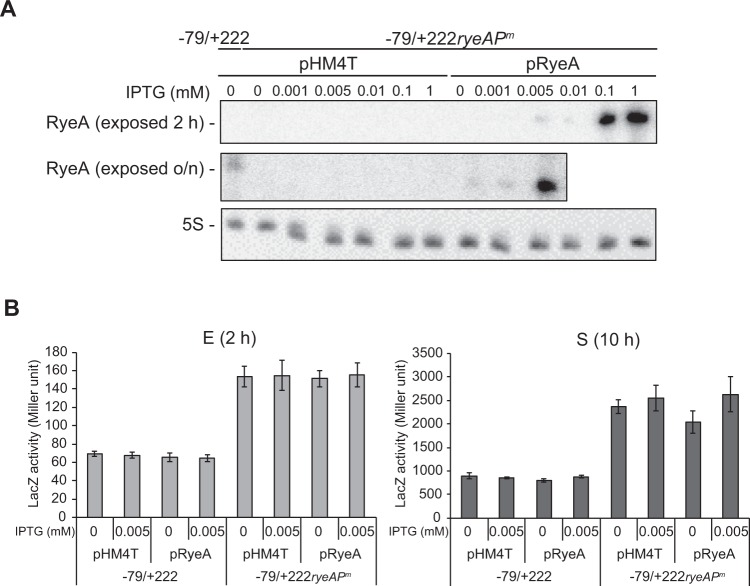
Effects of RyeA on *sdsR* transcription. (**A**) Ectopically expressed RyeA in DJ480Δ*ryeA/sdsR* cells carrying the *sdsR*(−379/+222)-*lacZ* or *sdsR*(−379/+222*ryeAP*^*m*^)-*lacZ* fusion were analyzed. Total cellular RNAs from pRyeA-containing cells induced with different IPTG concentration were analyzed by Northern blotting. pRyeA, RyeA-expressing plasmid derived from vector pHM4T. (**B**) LacZ activities were measured in cells ectopically expressing RyeA by induction with 0.005 mM IPTG. Exponential (**E**) and stationary (S) phase cells (grown for 2 h and 10 h, respectively) were used for LacZ assays (mean ± SD; n = 3).

**Figure 7:**
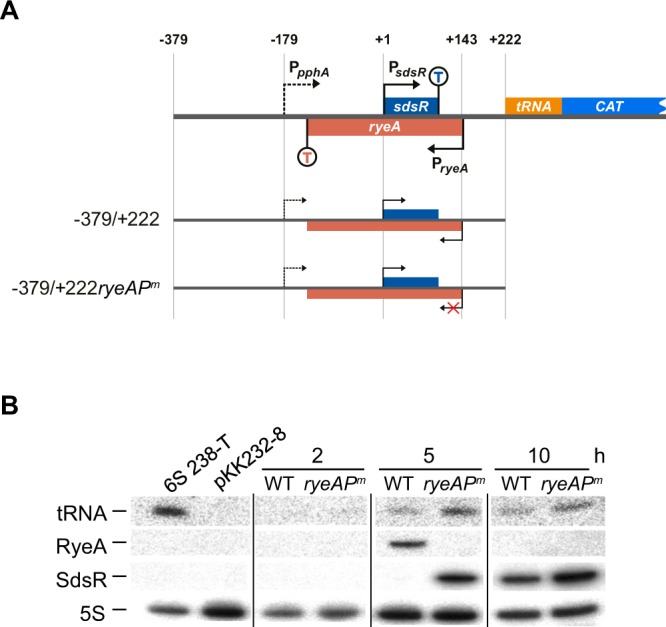
Analysis of read-through transcripts traversing the *sdsR* terminator. (**A**) Schematic representation of *sdsR*-tRNA^Arg^-CAT fusion plasmids. The *B*. *albidum* tRNA^Arg^ gene was inserted in the region before the CAT gene of pKK232-8 plasmid. (**B**) Northern blot analysis of Arg-tRNA, RyeA, and SdsR. Total cellular RNA from cells carrying the *sdsR*(−379/+222)-tRNA^Arg^-CAT or *sdsR*(−379/+222*ryeAP*^*m*^)-tRNA^Arg^-CAT fusion plasmid were isolated and analyzed by Northern blotting. In each panel the spliced images from the same Northern membrane are shown with the insertion of a dividing lines between the spliced lanes.

Next, we examined whether *ryeA* transcription affected *pphA* expression using the *ryeA* promoter mutant. To avoid possible effects of chromosomally expressed SdsR and RyeA, we constructed the *sdsR*-*lacZ* fusions in an *sdsR/ryeA*-knockout background. The LacZ activity of the *sdsR*(−379/+222)-*lacZ* fusion was increased by about 4-fold by the *ryeA* promoter mutation (Fig. 5). This increase could be attributable to the absence of RyeA (acting in *trans*) or *ryeA* transcription itself (acting in *cis*). To discriminate between these two possibilities, we analyzed LacZ activity following ectopic expression of RyeA. To determine conditions for ectopically expressing RyeA at levels comparable to those generated by the endogenous *ryeA* promoter, we varied the concentration of isopropyl β-D-1-thiogalactopyranoside (IPTG) used to induce expression (Fig. 6A). We found that RyeA levels induced by 0.005 mM IPTG were comparable to those produced by the *ryeA* promoter. Induction of RyeA caused no decrease in LacZ activity (Fig. 6B), suggesting that the absence of RyeA is not responsible for the increase in LacZ activity. Therefore, it is likely that *ryeA* transcription, not RyeA, inhibits *pphA* expression by reducing read-through transcription from the *sdsR* promoter. Then we examined how *ryeA* transcription affects the read-through transcription during the growth using the *sdsR(*−*379/*+*222)*-*lacZ* fusion. We found that the *ryeA* transcription represses LacZ expression at all growth phases and delays its expression 3 h to the stationary phase (Fig. S5). To further confirm that read-through transcripts are increased by the *ryeA* mutation, we inserted a *Brevibacterium albidum* tRNA^Arg^ sequence between the *sdsR* sequences and the CAT coding sequence in the *sdsR*-CAT fusion constructs and examined exogenous tRNA expression as well as SdsR in an *sdsR/ryeA*-knockout background (Fig. 7). The reason that we used heterologously expressed *B*. *albidum* tRNA^Arg^, was because the tRNA was previously shown to be metabolically stable in *E*. *coli* and detectable by Northern blot analysis without cross-hybridization with *E*. *coli* tRNAs^36^. The *ryeA* promoter mutation caused an increase in tRNA^Arg^ expression, confirming that the *ryeA* mutation increases read-through transcription. Taken together, these data show that *pphA* expression is inhibited by *ryeA* transcription itself, and not by RyeA.

**Figure 8:**
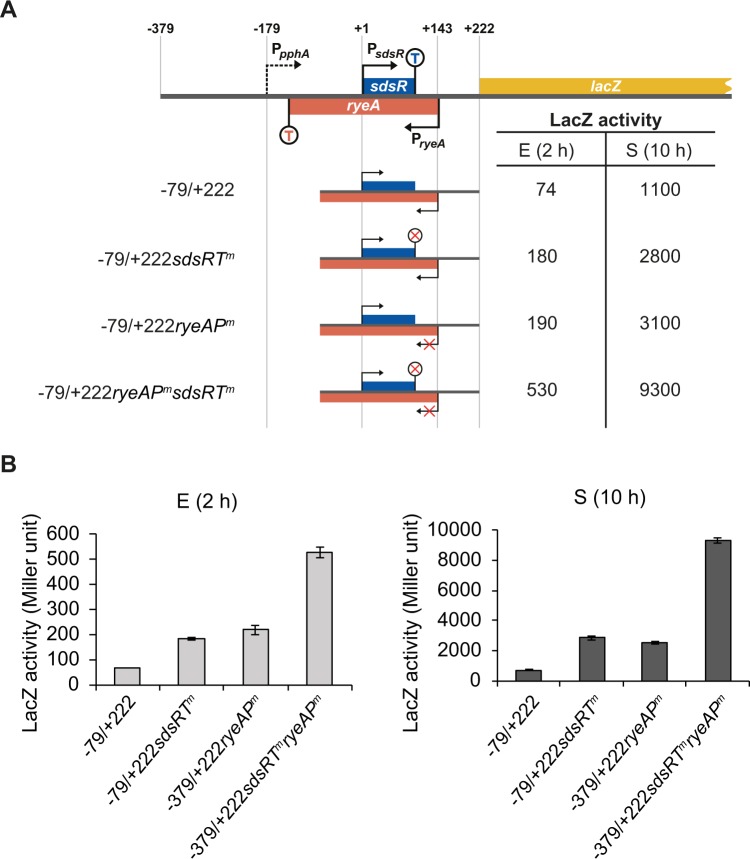
*sdsR* terminator-independency of transcriptional interference between the *sdsR* and *ryeA* promoter. (**A**) Schematic diagrams of *sdsR*(−79/+222)-*lacZ* transcriptional fusion constructs are shown along with their LacZ activities for DJ480Δ*ryeA/sdsR* cells grown for 2 h (**E**) and 10 h (S) post-inoculation at 37° (**B**) The LacZ activities are presented in bar graph. *sdsRT*^*m*^, *sdsR* terminator mutation; *ryeAP*^*m*^, *ryeA* −10 promoter mutation (mean ± SD; n = 3).

To examine whether the increase in read-through transcripts was caused by reduced termination efficiency at the *sdsR* terminator, we introduced a terminator mutation into the *sdsR*(−79/+222)-*lacZ* fusion (Fig. 8). This terminator mutation did not affect the *ryeA* promoter mutation-induced increase in LacZ activity, suggesting that the increase in read-through transcripts caused by the *ryeA* promoter mutation does not result from reduced termination efficiency. Therefore, it seems likely that transcription from the *ryeA* promoter interferes with transcription of *sdsR*.

Because it is known that transcriptional interference can occur in DNA constructs with convergent promoters^37^, we examined the existence of transcriptional interference with respect to *ryeA* transcription using *in vitro* transcription assays using pSdsR(−379/+222) carrying the *ryeA* promoter and its *ryeA* promoter mutant derivative, pSdsR(−379/+222*ryeAP*^*m*^) (Fig. 2). For these assays, we used different ratios of Eσ^70^ and Eσ^S^. As expected, the increased ratio of Eσ^S^/Eσ^70^ increased SdsR and its read-through transcripts (SdsR-*rnpB*) with the decrease of RyeA (Fig. 2B,C), which may be reminiscent of their reciprocal synthesis during growth. The *ryeA* promoter mutation increased both SdsR and read-through transcripts, producing the highest increase in levels at an Eσ^70^ to Eσ^S^ ratio of 1:9 (Fig. 2B), which may be similar to that in the stationary phase *in vivo*.

Transcriptional interference can be due to promoter occlusion, colliding RNA polymerases, or transcription-induced changes in DNA supercoiling that affect initiation of transcription. Promoter occlusion is not likely because the two promoters are far apart. We tested whether positive supercoiling induced by transcription from the *ryeA* promoter could affect transcription from the *sdsR* promoter. For this purpose, we treated cells with novobiocin, a gyrase subunit B inhibitor. Growth of *E*. *coli* cells was inhibited by novobiocin with a 50% inhibitory concentration (IC_50_) of 100 μg/ml (Fig. S6A). We examined effects of *ryeA* transcription on *sdsR* transcription in the presence of novobiocin at 100 μg/ml (Fig. S6B). Since SdsR levels could be affected by RyeA *in trans*, we analyzed the novobiocin effects in LacZ activity from *sdsR*(−379/+222)-*lacZ* fusion rather than those in SdsR levels. Novobiocin had little effect on the increase of LacZ activity by the *ryeA* mutation, suggesting that transcription-induced changes in DNA supercoiling contribute little to the observed transcriptional interference. On the other hand, when we examined the *ryeA* and *sdsR* promoter activities during growth using *lacZ* transcriptional fusions, the *ryeA* and *sdsR* promoters showed transcription activities in all the growth phases, although *ryeA* promoter is more active in the exponential phase than the *sdsR* promoter (Fig. S7). These results altogether suggest the possibility that two oppositely transcribing RNA polymerases collide in the cell. Therefore, it seems likely that *ryeA* transcription inhibits transcription from the *sdsR* promoter through transcriptional interference, which may occur by RNA polymerase collision sometime between RNA polymerase binding to the *sdsR* promoter and the transcription elongation step up until the RNA polymerase reaches the *ryeA* promoter by collision between oppositely transcribing RNA polymerases.

## Discussion

In this study, we first defined the transcription units and biogenesis of *sdsR* and *ryeA* in more detail, showing that 3′ ends of both SdsR and RyeA correspond to their own transcription termini. We identified three transcription initiation start sites from the *ryeA* promoter. The primary *ryeA* transcripts are trimmed at the 5′ end to form processed RNAs with 5′ monophosphate whose 5′ end corresponds to the most downstream transcription start, which can generate RyeA of 272 nt with the longest 3′ end. On the other hand, the 5′ end of SdsR, which is the same as the *sdsR* transcription initiation site, retains triphosphate. This may explain that RyeA has a much shorter half-life than SdsR in the cell^18^ because RNA with 5′ monophosphate is more vulnerable to degradation than RNA with 5′ triphosphate^38^. The transcription of *sdsR* terminates at base positions ranging from +100 to +102 relative to its own transcription start site, generating the longest SdsR (102 nt) in the cell.

RyeA is expressed in the exponential phase, whereas SdsR expression starts in late-stationary phase. Since we showed here that RyeA and SdsR are almost exclusively transcribed *in vitro* by Eσ^70^ and Eσ^S^, respectively, acting at their respective promoters, the growth-dependent regulation of RyeA and SdsR should be mediated by the sigma factor selectivity of the two promoters. Promoter mutation analyses revealed that mutual degradation of SdsR and RyeA also contributes to discrete growth phase-dependent regulation of the expression of each sRNA. Furthermore, RyeA transcription can interfere with SdsR transcription, generating less SdsR when RyeA transcription occurs. As a result of this biosynthetic pathway, more restricted stationary phase expression of SdsR can be achieved through the coupled action of degradation of SdsR by RyeA expressed in the exponential phase and inhibition of SdsR transcription through transcriptional interference by RyeA transcription from the σ^70^-dependent *ryeA* promoter.

RNA-seq analysis showed that expression of *pphA*, encoding a phosphatase, downstream of *sdsR* was decreased by the *sdsR* promoter mutation and increased by the *ryeA* promoter mutation, suggesting that *pphA* expression is controlled by the *sdsR/ryeA* regulatory circuit. We showed that *pphA* mRNA is transcribed as an *sdsR*-*pphA* dicistronic RNA from the σ^S^-dependent *sdsR* promoter rather than from the previously reported heat-inducible *pphA* promoter. Thus, *pphA* mRNA may not be transcribed from the heat-inducible promoter, as suggested previously^30^. However, *pphA* expression differs from SdsR expression in that it is not affected in *trans* by RyeA although the *ryeA* transcription represses *pphA* expression. Therefore, *pphA* could be expressed at an earlier growth phase than SdsR. *Trans*-acting sRNAs usually downregulate expression of multiple target genes by inhibiting translation initiation of mRNA or by inducing degradation^5^. Our RNA-seq analysis showed that the *sdsR* promoter mutation upregulated more than 100 genes, but the *ryeA* promoter mutation led to only a few upregulated genes, suggesting that a major function of RyeA is to downregulate SdsR as a *cis*-encoded sRNA. The finding that the well-known SdsR target gene *tolC*^22^ was repressed by the *ryeA* promoter mutation and activated by the *sdsR* mutation supports this possibility.

Our study showed that *pphA* expression begins with transcription from the *sdsR* promoter, and requires read-through transcription over the *sdsR* terminator and continued transcription into the *pphA* coding region. RNA-seq data showed that there was a considerable amount of read-through RNA over the *sdsR* terminator, but the amount of RNA continued to decrease with progression into the *pphA* open reading frame. Therefore, it is likely that other regulatory systems, such as premature termination or translational control, in addition to the *sdsR/ryeA* regulatory circuit are involved in *pphA* regulation. This remains to be determined in the future.

Antisense RNAs are expressed through convergent transcription, and their expression promotes transcriptional interference^37^. Transcriptional interference can come into play through various mechanisms, depending on the promoter strength and transcription velocity^39^. In this context, the *sdsR/ryeA* transcription unit is interesting because transcription levels from the *ryeA* and *sdsR* promoter vary during growth, leading to different transcriptional interference levels depending on growth conditions. It is likely that the *sdsR/ryeA* region at which transcriptional interference between two RNA polymerases occurs depends on the growth phase. SdsR transcription can be affected by convergent *ryeA* transcription at both initiation and elongation steps because the entire *sdsR* transcription unit is encompassed by the *ryeA* gene. Like SdsR expression, *pphA* expression is also affected by transcriptional interference by *ryeA* transcription.

**Figure 9:**
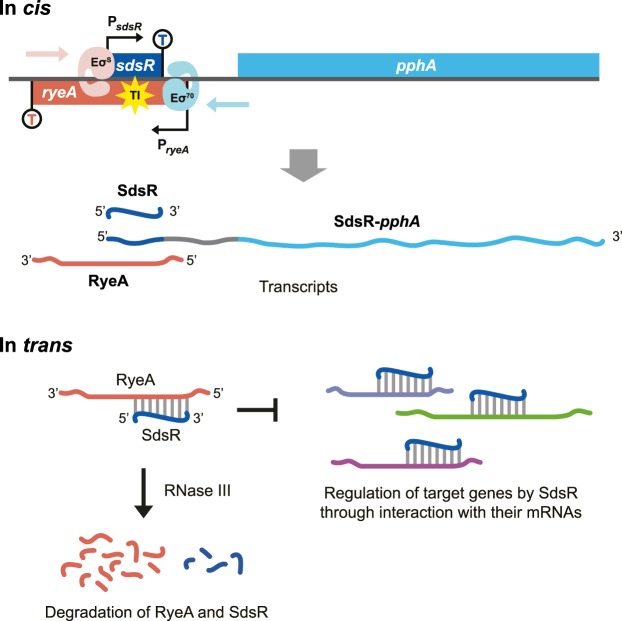
Schematic model of the *sdsR/ryeA* regulatory circuit. During transcription of RyeA, SdsR and dicistronic *sdsR*-*pphA* mRNA, transcriptional interference (TI) occurs in *cis* through collision of Eσ^70^ with Eσ^S^. RyeA and SdsR bind each other and the RyeA and SdsR duplex is degraded with the help of RNase III. This results in inhibition of SdsR regulation of target gene mRNA expression.

To summarize, we herein characterized regulation of the expression of the toxin SdsR, including its downstream *pphA* gene, and the antitoxin RyeA (Fig. 9). SdsR expression is tightly regulated during growth through transcription from the σ^S^-dependent *sdsR* promoter, RyeA-mediated degradation of SdsR, and transcriptional interference from the σ^70^-dependent *ryeA* promoter. On the other hand, downstream *pphA* expression, like that of SdsR, is also under control of both *sdsR* and *ryeA* promoters, but is unaffected by RyeA. Therefore, the *sdsR/ryeA* regulatory circuit plays a critical role in tightly controlling growth-dependent expression of SdsR toxin and *pphA* to ensure that they are not expressed during the exponential phase.

## Experimental Procedures

### Strains, plasmids, and oligonucleotides

All strains and plasmids used in this study are listed in Table 2. All primers and oligonucleotides used in this study are shown in Table 3. To generate *sdsR* and *ryeB* promoter mutant strains, each −10 element was changed to ‘CTCGAG’ using scarless mutagenesis, as described previously^40^. A series of lysogen-containing *lacZ* transcriptional fusion constructs was prepared. Briefly, various promoter regions around *ryeA/sdsR* were amplified, and the resulting fragment was cloned between the *Eco*RI and *Bam*HI sites of the pRS1553 vector to generate *lacZ* transcriptional fusion plasmids. Lysogens were constructed by transforming *E*. *coli* strain DJ480 with the various fusion plasmids and transfecting with λRS468 to construct the corresponding *lacZ* fusion lysogens. Single-copy integration was confirmed by PCR^41^. For point mutations in *sdsR*-*lac*Z fusions, site-directed mutagenesis was performed, as described previously^18^. DJ480Δ*sdsR/ryeA* strain was generated from MG1655Δ*sdsR/ryeA* by P1-mediated transduction^42^ and confirmed by sequence analysis of the amplified, knocked-out region. pRyeA and pSdsR carrying a pBR322 origin ectopically express IPTG-inducible RyeA and SdsR, respectively^43^. Plasmid pAKA-ara containing a pACYC184 origin was constructed by cloning the pBAD-AraC DNA sequence into the *Ava*I/*Eco*RI sites of plasmid pAKA, and used as a cloning vector to generate pRyeA-ara and pSdsR-ara, as described previously^44^, which express arabinose-inducible RyeA and SdsR, respectively. To generate *sdsR*- or *ryeA*-CAT fusion plasmids, *sdsR* or *ryeA* promoter-containing DNA fragments were obtained via PCR amplification of genomic DNA. The resulting PCR products were digested with *Bam*HI/*Hin*dIII and ligated into pKK232–8. To generate *sdsR*-tRNA^Arg^-CAT fusion plasmids, a *B*. *albidum* tRNA^Arg^ sequence^36,45^ was amplified by PCR and inserted immediately upstream of the CAT gene in the *sdsR*-CAT fusion plasmids. Template DNA plasmids for *in vitro* transcription were prepared by cloning *sdsR* or *ryeA* promoter-containing DNA fragments into the *Hin*dIII/*Eco*RI site of plasmid pLS16^35^. The oligonucleotides employed are listed in Table 3.

**Table 2:** Strains and plasmids used in this study.

Name	Description	Source
***Strains***		
MG1655	*E*. *coli* MG1655 wild type	Laboratory stock
*ryeAP* ^*m*^	MG1655 chromosomal *ryeA* promoter mutant (mutation on -10 promoter substituted to Xho1 site)	This study
*sdsRP* ^*m*^	MG1655 chromosomal *sdsR* promoter mutant (mutation on -10 promoter substituted to Xho1 site)	This study
MG1655Δ*sdsR/ryeA*	MG1655Δ*ryeA/sdsR::kan*^*R*^	^18^
DJ480	*E*. *coli* DJ480 wild type used for *lacZ* fusion	^51^
DJ480Δ*sdsR/ryeA*	P1 transduction from MG1655Δ*sdsR/ryeA*	This study
***Plasmids***		
pWRG99	Plasmid carrying temperature-sensitive λ-red recombinase and I-SceI endonuclease	^40^
pWRG100	Template DNA plasmid for constructing scarless mutants	^40^
pKK232-8	A plasmid encoding *cat* gene encoding cam acetyltransferase (CAT) for chloramphenicol resistance	^52^
p22A	A derivative of pGEM3 carrying *B*. *albidum* tRNA^Arg^	^36, 45^
pRS1553	A cloning vector for construction of *lacZ* transcriptional fusion	^43^
pHM4T	A derivative of pHM1, Amp^R^, IPTG-inducible RNA expression vector	^44^
pRyeA	A deriavative of pHM4T carrying *ryeA* full sequence, with a mutation on -10 promoter element of *sdsR*.	^18^
pSdsR	A deriavative of pHM4T carrying *sdsR* full sequence.	^18^
pLS16	*In vitro* transcription template vector	^44^
pAKA-ara	A derivative of pAKA, Tc^R^, arabinose-inducible RNA expression vector	This study
pRyeA-ara	A deriavative of pAKA-ara carrying *ryeA* full sequence, with a mutation on -10 promoter element of *sdsR*.	This study
pSdsR-ara	A derivative of pAKA-ara carrying *sdsR* full sequence	This study

**Table 3:** Oligonucleotide sequences.

Name	Sequence 5′-3′	Use
RyeA-p1	GCGGCGCAACTGCTCAAGACAACC	Chromosomal *ryeA* -10 promoter mutant cloning
RyeA-p2	TCACTCGAGACACACACTCTACTG	Chromosomal *ryeA* -10 promoter mutant cloning
RyeA-p3	TGTGTGTCTCGAGTGACTATAAAG	Chromosomal *ryeA* -10 promoter mutant cloning
RyeA-p4	CAAACTGGAAAACCTGGCGTCGTC	Chromosomal *ryeA* -10 promoter mutant cloning
SdsR-p1	GCGGCGCAACTGCTCAAGACAACC	Chromosomal *sdsR* -10 promoter mutant cloning
SdsR-p2	TCACTCGAGACACACACTCTACTG	Chromosomal *sdsR* -10 promoter mutant cloning
SdsR-p3	TGTGTGTCTCGAGTGACTATAAAG	Chromosomal *sdsR* -10 promoter mutant cloning
SdsR-p4	CAAACTGGAAAACCTGGCGTCGTC	Chromosomal *sdsR* -10 promoter mutant cloning
SdsR-pRcom1	ATAGCCTTTTGATTTCCAAT	Chromosomal *sdsR* -10 promoter mutant cloning
SdsR-pRcom2	CTGTATTCGGTCCAGGGAAA	Chromosomal *sdsR* -10 promoter mutant cloning
5RACE_F	GATATGCGCGAATTCCTGTAGAAC	5′ RACE primer
5RACE_RyeAR	CCGCCTCAGTTCTTTCACCA	5′ RACE GSP1 primer
5RACE_SdsRR		5′ RACE GSP1 primer
5RACE_Nested	CCTGTAGAACGAACACTAGAAG	5′ RACE primer
5RACE_Nested_RyeAR	GCAAGGCAACTAAGCCTGCA	5′ RACE GSP2 primer
5RACE_Nested_SdsRR	TCGGTCCAGGGAAATGGCTC	5′ RACE GSP2 primer
3RACE_R	GAGCATGCGGCCGCTAAGAACAGT	3′ RACE primer
3RACE_RyeAF	AAAGTCAGCGAAGGAAATGC	3′ RACE primer
3RACE_SdsRF	GCAAGGCAACTAAGCCTGCA	3′ RACE primer
3RACE_Nested_RyeAF	CACGATTCCTGTATTCGGTC	3′ RACE primer
3RACE_Nested_R	CGGCCGCTAAGAACAGTGAA	3′ RACE primer
AvaIAraCF	CCCTCGGGCTGGCCCCG	pBAD-AraC cloning into pAKA
AraCREcoRI	CGGAATTCGTT TGGCCAAACA GTAGAGAG	pBAD-AraC cloning into pAKA
HD3pphAPF	CCCAAGCTTGCGAAGAGGTGATGCTG	−379 +222 cloning into pLS16
ERIpphAR	CGGAATTCGCGCTAAAGATCACATAATC	−379 +222 cloning into pLS16
BamHISdsRPF	CGGGATCCAGCTGATGACCACC	*sdsR*-CAT fusion
HinDIIISdsRPR	CCCAAGCTTAGTTGCCTTGCCC	*sdsR*-CAT fusion
HINDIIIryeAPF	CCCAAGCTTCGCTGACTTTATAGTCAACC	*ryeA*-CAT fusion
BAMHIryeAPR	CGGGATCCTTGCGGCGCAACTGC	*ryeA*-CAT fusion
BAMHI-379PF	CGGGATCCGCGAAGAGGT GATG	*sdsR*(-379 +131)-CAT fusion
Hind3 + 131R	CCCAAGCTTAAGGAAATGC TTCTGGC	*sdsR*(-379 +131)-CAT fusion
tRNA_F	TACAAGCTTGCGCTCGTAGCTCAG	*sdsR*-tRNA^Arg^-CAT fusion
tRNA_R	TATAAGCTTTGGTGCGCCCGGCGG	*sdsR*-tRNA^Arg^-CAT fusion
ERI-379F	CGGAATTCGCGAAGAGGTGATG	*sdsR*-*lacZ* fusion
ERI-79F	CGGAATTCAGCTGATGACCACC	*sdsR*-*lacZ* fusion
ERI + 104F	CGGAATTCATCTGTTAAAAGCCAGAAGC	*sdsR*-*lacZ* fusion
ERI + 142F	CGGAATTCTAGTCAACCATAACACACACT	*sdsR*-*lacZ* fusion
ERIA-80F	CGGAATTCCGCTAAAGATCACATAATC	*ryeA*-*lacZ* fusion
BHI-170R	CGGGATCCGTCATCAGCTGAAATG	*sdsR*-*lacZ* fusion
BHI + 11R	CG GGATCCAGTTGCCTTGCCC	*sdsR*-*lacZ* fusion
BHI + 222R	CGGGATCCGCGCTAAAGATCACATAATC	*sdsR*-*lacZ* fusion
BHI + 237R	CGGGATCCCAATATTTTCCTCCCGCGCT	*sdsR*-*lacZ* fusion
BHIA + 10R	CGGGATCCCGCTGACTTTATAGTCAACC	*ryeA*-*lacZ* fusion
CAT_R	GGTGGTATATCCAGTGATTTTTTTC	Primer extension primer for RyeA and SdsR
SdsR + 59R	GCTCTTGGGA GAGAGCCG	Primer extension primer for *pphA*
RyeA_NP	CGTGTTCGGTCTCTTTTTATCTG	RyeA Northern probe
SdsR_NP	CGTGCGCTAAAAGTTGGCATTAATGC	SdsR Northern probe
5SRNA + 90R	GAGACCCCACACTACCATCGG	5S RNA Northern probe
tRNA_NP	GAAACCTGTACTCTATCCAACTGAGCTA	tRNA Northern probe
pphA_F	TATCAGAGAATTGCGGGTCATC	*pphA* mRNA qRT-PCR
pphA_R	ACGTCTCCCACTGAGATAAGTA	*pphA* mRNA qRT-PCR
asnA_F	GATGGCACGCAGGATAACT	*asnA* mRNA qRT-PCR
asnA_R	CTGAAGTCGTGTTGCCCTAA	*asnA* mRNA qRT-PCR
aphA_F	GCAAGATCACACAGGCAATC	*aphA* mRNA qRT-PCR
aphA_R	CGACCGAAACCCAATGAATG	*aphA* mRNA qRT-PCR
stpA_F	CGCGTCCGGCGAAATATAA	*stpA* mRNA qRT-PCR
stpA_R	AGATCAGGAAATCGTCGAGAGA	*stpA* mRNA qRT-PCR
ytfE_F	GTGAACTGGCGCTCTCTATT	*ytfE* mRNA qRT-PCR
ytfE_R	GGTTGTTCAGCGAGCTTTG	*ytfE* mRNA qRT-PCR
ynfF_F	TCAGGTTCGAGCGTGTTTAC	*ynfF* mRNA qRT-PCR
ynfF_R	GGGCTTCGTCCCAACTTATC	*ynfF* mRNA qRT-PCR
tolC_F	TAG TAA CCC GGA ATT GCG TAA G	*tolC* mRNA qRT-PCR,^18^
tolC_R	AGC CGT TGC TAT AGG TGT AAT C	*tolC* mRNA qRT-PCR,^18^
putA_F	GGCCAATGAGAGCGATGAA	*putA* mRNA qRT-PCR
putA_R	GCTTGTTCCAGCAGCATAGA	*putA* mRNA qRT-PCR
gapA_F	GCACCACCAACTGCCTGGCT	*gapA* mRNA qRT-PCR,^50^
gapA_R	CGCCGCGCCAGTCTTTGTGA	*gapA* mRNA qRT-PCR,^50^
PM_RyeAF	CGCTGACTTTATAGTCACTCGAGACACACACTCTACTGTC	*ryeA* promoter mutation
PM_RyeAR	TAACAAGAATGTTAAAAAACGCTGGACTCAGACAGTAGAGTGTGTGTC	*ryeA* promoter mutation
SdsRtMF	CATTTCCCTGGAGGCAATACAG GAATCGTGTT	*sdsR* terminator mutation
SdsRtMR	TCCAGGGAAATGGCTCTTGGGAGAGAGCCGTG	*sdsR* terminator mutation
PM_SdsRF	CCTTTACTCGAGGATGACGACGCCAG	*sdsR* promoter mutation
PM_SdsRR	GTCATCCTCGAGTAAAGGGCAAGGCAAC	*sdsR* promoter mutation

### Primer extension

An SdsR +59R primer (5′-GCT CTT GGG AGA GAG CCG-3′) was 5′ end-labeled with [γ-^32^P]ATP using T4 polynucleotide kinase. The primer was then used to analyze *sdsR*(−379/+131)-CAT fusion transcripts. Total cellular RNA was isolated from cells carrying the *sdsR*(−379/+131-CAT fusion plasmid. The labeled primer was used for primer extension analysis, as previously described^46^.

### RACE assays

5′ and 3′ RACE analysis for RyeA and SdsR were performed as described previously^47^. Total cellular RNAs from mid-exponential phase (4 h) and stationary phase (8 h) cells were used for RyeA and SdsR, respectively. For 5′ RACE total cellular RNA was treated with 5 unit of *E*. *coli* RppH (New England Biolabs) in a 50 μl reaction before RNA ligation.

### *In vitro* transcription by *E. coli* RNA polymerase

*In vitro* transcription using *E coli* RNA polymerase was performed as described previously^46^. *E*. *coli* RNA polymerase holoenzyme Eσ^70^ was purchased from New England Biolabs. σ^S^ (RpoS) was purified using the plasmid described previously^46^. Eσ^S^ was reconstructed by combining the core enzyme (New England Biolabs) and σ^s^ in a molar ratio of 1:2. After 1 h reaction at 37 °C, the reaction was terminated by adding phenol/chloroform.

### Northern blotting

*E*. *coli* cells were grown overnight in LB broth in the presence of appropriate antibiotics. Overnight cultures were diluted 1:100 in fresh LB medium and further cultured at 37 °C. Total cellular RNA was extracted at the desired time points using the acidic hot-phenol method, as described previously^35^. RNA was generated *in vitro* using the T7 RiboMAX Express Large Scale RNA Production System (Promega). Northern blot analysis was carried out as described previously^35^. Briefly, 5–10 μg of total RNA was fractionated on a 5% polyacrylamide gel containing 7 M urea and electrotransferred to a Hybond-XL membrane (Amersham Biosciences). Membranes were hybridized with ^32^P-labeled DNA probes in PerfectHyb Plus Hybridization Buffer (Sigma Aldrich) and analyzed using an FLA 7000 Image Analyzer (Fuji).

### Mutual degradation assays of SdsR and RyeA

MG1655*ΔsdsR/ryeA* cells were co-transformed with a plasmid pair of pSdsR-ara/pRyeA or pRyeA-ara/pSdsR. Overnight cultures of the transformed cells were diluted (1:100) into LB medium containing ampicillin (100 μg/ml) and tetracycline (10 μg/ml), and grown at 37 °C for 2 h. Arabinose of 0.2% was added into the culture and cells were grown for 5 min or 25 min. Then 0.1 mM IPTG was added into each culture and cells were further grown further. Aliquots of the cell culture were taken in intervals and their RyeA and SdsR contents were analyzed by Northern blotting

### RNA-seq sample preparation and analysis

*E*. *coli* MG1655 wild-type, *ryeA* promoter mutant, and *sdsR* promoter mutant cells were grown at 37 °C overnight. After diluting 1:100, overnight cultures were grown for 3 h (exponential phase) and 8 h (stationary phase); total RNA was extracted at each point. All preparation procedures were as previously described^18^. All RNA sequencing and alignment procedures were conducted by ChunLab. Ribosomal RNA was depleted using a Ribo-Zero rRNA removal kit (Epicentre) according to the manufacturer’s instructions. Libraries for Illumina sequencing were generated using a TruSeq Stranded mRNA Sample Prep kit (Illumina) according to the manufacturer’s protocol. RNA sequencing was performed on the Illumina HiSeq 2500 platform using single-end 50-bp sequencing. Sequence data for the reference genome were retrieved from the NCBI database. Quality-filtered reads were aligned to the reference genome sequence using Bowtie2.

Transcript abundance was measured as Relative Log Expression (RLE). To screen for mRNAs whose levels differed by more than 2-fold versus wild-type cells, we filtered for mRNAs with EdgeR p-values < 0.05. For GO analyses, the 171 genes and 55 genes found to be up- or downregulated in the exponential phase and stationary phase, respectively, by the *sdsR* promoter mutation were sorted according to their GO categories (http://amigo.geneontology.org)^48^.

### LacZ assay

Three colonies for each strain were cultured overnight in LB medium with/without ampicillin (100 μg/ml), after which overnight cultures were diluted 1:100 and cultured in fresh medium containing 0.02% arabinose and 1 mM IPTG. Cultures were incubated for 2 h for exponential phase and 10 h for stationary phase. LacZ activity was assayed as described previously^49^.

### Semi-quantitative RT-PCR and qRT-PCR

RNA extraction and DNase treatment were performed as described above for RNA-seq. cDNA was synthesized using M-MLV reverse transcriptase (Enzynomics) with specific primers for semi-quantitative RT-PCR. qRT-PCR was performed on an Exicycler 96 system (Bioneer) using Prime Q-master mix (Genet Bio). Expression levels of each mRNA of interest were normalized to that of the *gapA* gene^50^. All experiments were performed according to the manufacturer’s instructions.

## Supplementary information

Supplementary information

## Data Availability

RNA-seq raw data for this study have been deposited in the National Center for Biotechnology Information Gene Expression Omnibus (GEO) and are accessible through the GEO Series accession number GSE122921.
