# Supplementary material for: Coordinate regulation of the expression of SdsR toxin and its downstream pphA gene by RyeA antitoxin in Escherichia coli
Source: Sci Rep. 2019 Jul 3;9:9627. doi: 10.1038/s41598-019-45998-z (PMC6610125; doi:10.1038/s41598-019-45998-z)
Supplement: Supplementary file 1 — Supplementary information [file 41598_2019_45998_MOESM1_ESM.docx]

**Supplementary Information**

**Coordinate regulation of the expression of SdsR toxin and its downstream *pphA* gene by RyeA antitoxin in *Escherichia coli***

Jee Soo Choi, Hongmarn Park, Wonkyong Kim and Younghoon Lee*

*Department of Chemistry, KAIST, Daejeon 34141, Korea*

Key words: *E. coli*, sRNAs, SdsR, RyeA, *pphA*, transcriptional interference

*Corresponding author

E-mail: Younghoon.Lee@kaist.ac.kr

Fax: +82-42-350-2810

Phone: +82-42-350-2872

**Table S1. RNA-seq analysis of mRNAs upregulated and downregulated over two-fold in *ryeA* promoter mutant cells at exponential phase (3 h)**

| **Upregulated** | | | **Downregulated** | | |
| --- | --- | --- | --- | --- | --- |
| **No.** | **Gene name** | **Fold change**  **(WT vs *ryeAP^m^*)^a^** | **No.** | **Gene name** | **Fold change**  **(WT vs *ryeAP^m^*)^a^** |
| **1** | *yrbN* | 3.05 | **1** | *ryeA* | -6.62 |
| **2** | *ysaC* | 2.16 | **2** | *leuL* | -2.54 |
| **3** | *ybcN* | 2.05 | **3** | *yibV* | -2.28 |
| **4** | *wcaD* | 1.63 | **4** | *asnA* | -1.62 |
| **5** | *gspH* | 1.40 | **5** | *omrA* | -1.62 |
| **6** | *yjbG* | 1.37 | **6** | *ygeH* | -1.39 |
| **7** | *mtr* | 1.15 | **7** | *puuD* | -1.28 |
|  |  |  | **8** | *eutS* | -1.26 |
|  |  |  | **9** | *tfaQ* | -1.14 |
|  |  |  | **10** | *intG* | -1.11 |

^a^Fold changes for each mRNA are in log2 scale in the wild type (WT) versus *ryeA* promoter mutant (*ryeAP^m^*) comparison.

**Table S2. RNA-seq analysis of mRNAs upregulated and downregulated over two-fold in *ryeA* promoter mutant cells at stationary phase (8 h)**

| **Upregulated** | | | **Downregulated** | | |
| --- | --- | --- | --- | --- | --- |
| **No.** | **Gene name** | **Fold change**  **(WT vs *ryeAP^m^*)^a^** | **No.** | **Gene name** | **Fold change**  **(WT vs *ryeAP^m^*)^a^** |
| **1** | *sdsR* | 2.68 | **1** | *tfaQ* | -3.29 |
| **2** | *yibV* | 1.05 | **2** | *intG* | -1.86 |
|  |  |  | **3** | *aphA* | -1.09 |

^a^Fold changes for each mRNA are in log2 scale in the wild type (WT) versus *ryeA* promoter mutant (*ryeAP^m^*) comparison.

**Table S3. RNA-seq analysis of mRNAs upregulated over two-fold in *sdsR* promoter mutant cells at exponential phase (3 h)**

| **No.** | **Gene name** | **Fold change**  **(WT vs *sdsRP^m^*)^a^** | **No.** | **Gene name** | **Fold change**  **(WT vs *sdsRP^m^*)^a^** | **No.** | **Gene name** | **Fold change**  **(WT vs *sdsRP^m^*)^a^** |
| --- | --- | --- | --- | --- | --- | --- | --- | --- |
| **1** | *ytfE* | 6.13 | **41** | *cysN* | 2.84 | **81** | *norW* | 1.82 |
| **2** | *narH* | 5.93 | **42** | *nrfA* | 2.80 | **82** | *feoA* | 1.81 |
| **3** | *narG* | 5.72 | **43** | *ynfO* | 2.80 | **83** | *tdcA* | 1.78 |
| **4** | *narI* | 5.69 | **44** | *nirC* | 2.79 | **84** | *blr* | 1.76 |
| **5** | *hcp* | 5.60 | **45** | *ybcW* | 2.78 | **85** | *exbB* | 1.75 |
| **6** | *isrC* | 5.46 | **46** | *hmp* | 2.75 | **86** | *gsiA* | 1.74 |
| **7** | *nirB* | 5.45 | **47** | *garK* | 2.72 | **87** | *pagP* | 1.68 |
| **8** | *narJ* | 5.39 | **48** | *moaB* | 2.70 | **88** | *cspA* | 1.67 |
| **9** | *narK* | 5.14 | **49** | *azuC* | 2.68 | **89** | *ydjN* | 1.65 |
| **10** | *norV* | 4.80 | **50** | *ygaQ* | 2.64 | **90** | *cedA* | 1.63 |
| **11** | *cysI* | 4.67 | **51** | *yhiS* | 2.64 | **91** | *nrfF* | 1.61 |
| **12** | *cysW* | 4.50 | **52** | *nrfB* | 2.64 | **92** | *gudD* | 1.58 |
| **13** | *cysU* | 4.49 | **53** | *napH* | 2.63 | **93** | *yciW* | 1.52 |
| **14** | *cysJ* | 4.37 | **54** | *moaC* | 2.61 | **94** | *ttdR* | 1.50 |
| **15** | *napF* | 4.16 | **55** | *pinQ* | 2.58 | **95** | *ylcI* | 1.50 |
| **16** | *garL* | 4.14 | **56** | *moaA* | 2.58 | **96** | *yeiS* | 1.48 |
| **17** | *yoaG* | 4.07 | **57** | *yeeS* | 2.55 | **97** | *sbp* | 1.46 |
| **18** | *cysA* | 3.88 | **58** | *yoeF* | 2.52 | **98** | *dcuC* | 1.44 |
| **19** | *cysH* | 3.87 | **59** | *moaD* | 2.49 | **99** | *malT* | 1.42 |
| **20** | *yeeE* | 3.82 | **60** | *napB* | 2.42 | **100** | *dsbE* | 1.41 |
| **21** | *flu* | 3.80 | **61** | *fdnH* | 2.37 | **101** | *nth* | 1.40 |
| **22** | *cysD* | 3.77 | **62** | *napA* | 2.36 | **102** | *stpA* | 1.39 |
| **23** | *yeaR* | 3.70 | **63** | *napG* | 2.36 | **103** | *ymiA* | 1.39 |
| **24** | *napD* | 3.68 | **64** | *cnu* | 2.32 | **104** | *ccmE* | 1.39 |
| **25** | *nirD* | 3.63 | **65** | *fdnI* | 2.21 | **105** | *ccmH* | 1.37 |
| **26** | *gudX* | 3.46 | **66** | *fdnG* | 2.20 | **106** | *mlc* | 1.36 |
| **27** | *cysC* | 3.45 | **67** | *quuD* | 2.19 | **107** | *nrfD* | 1.35 |
| **28** | *yeeD* | 3.32 | **68** | *gudP* | 2.18 | **108** | *cysK* | 1.35 |
| **29** | *garR* | 3.17 | **69** | *bfd* | 2.14 | **109** | *ychO* | 1.33 |
| **30** | *cysP* | 3.15 | **70** | *ccmB* | 2.13 | **110** | *ccmC* | 1.32 |
| **31** | *yccM* | 3.12 | **71** | *yghF* | 2.13 |  |  |  |
| **32** | *garP* | 3.11 | **72** | *narX* | 2.12 |  |  |  |
| **33** | *ybgD* | 3.11 | **73** | *nrfC* | 2.07 |  |  |  |
| **34** | *yqeI* | 3.03 | **74** | *narL* | 2.07 |  |  |  |
| **35** | *insA* | 2.94 | **75** | *napC* | 2.04 |  |  |  |
| **36** | *ygbA* | 2.92 | **76** | *ccmA* | 2.04 |  |  |  |
| **37** | *yeeR* | 2.88 | **77** | *abrB* | 1.95 |  |  |  |
| **38** | *moaE* | 2.87 | **78** | *yrbN* | 1.90 |  |  |  |
| **39** | *hcr* | 2.85 | **79** | *manX* | 1.89 |  |  |  |
| **40** | *garD* | 2.85 | **80** | *ydjO* | 1.89 |  |  |  |

^a^Fold changes for each mRNA are in log2 scale in the wild type (WT) versus *sdsR* promoter mutant (*sdsRP^m^*) comparison.

**Table S4. RNA-seq analysis of mRNAs downregulated over two-fold in *sdsR* promoter mutant cells at exponential phase (3 h)**

| **No.** | **Gene name** | **Fold change**  **(WT vs *sdsRP^m^*)^a^** | **No.** | **Gene name** | **Fold change**  **(WT vs *sdsRP^m^*)^a^** |
| --- | --- | --- | --- | --- | --- |
| **1** | *ynfF* | -4.06 | **41** | *adk* | -1.45 |
| **2** | *ynfG* | -3.71 | **42** | *ryeA* | -1.45 |
| **3** | *ynfH* | -3.40 | **43** | *msrB* | -1.44 |
| **4** | *ydhX* | -2.88 | **44** | *yidC* | -1.43 |
| **5** | *hcaR* | -2.64 | **45** | *yfeK* | -1.43 |
| **6** | *ompW* | -2.63 | **46** | *csiD* | -1.41 |
| **7** | *fumB* | -2.55 | **47** | *mglC* | -1.38 |
| **8** | *ydhW* | -2.45 | **48** | *yliE* | -1.37 |
| **9** | *ydhV* | -2.43 | **49** | *yfcC* | -1.34 |
| **10** | *dctA* | -2.41 | **50** | *metK* | -1.32 |
| **11** | *psuK* | -2.34 |  |  |  |
| **12** | *dmsD* | -2.27 |  |  |  |
| **13** | *ydhU* | -2.22 |  |  |  |
| **14** | *ycgZ* | -2.13 |  |  |  |
| **15** | *ydhT* | -2.13 |  |  |  |
| **16** | *sdhC* | -2.01 |  |  |  |
| **17** | *insA* | -2.01 |  |  |  |
| **18** | *sdhD* | -2.00 |  |  |  |
| **19** | *glcC* | -1.99 |  |  |  |
| **20** | *mglA* | -1.93 |  |  |  |
| **21** | *astC* | -1.93 |  |  |  |
| **22** | *ynfE* | -1.92 |  |  |  |
| **23** | *tdcD* | -1.88 |  |  |  |
| **24** | *ugpB* | -1.86 |  |  |  |
| **25** | *ygiM* | -1.80 |  |  |  |
| **26** | *argT* | -1.70 |  |  |  |
| **27** | *fadB* | -1.66 |  |  |  |
| **28** | *galS* | -1.64 |  |  |  |
| **29** | *mhpR* | -1.64 |  |  |  |
| **30** | *tdcC* | -1.64 |  |  |  |
| **31** | *ndk* | -1.63 |  |  |  |
| **32** | *phoH* | -1.63 |  |  |  |
| **33** | *dcuB* | -1.62 |  |  |  |
| **34** | *mglB* | -1.61 |  |  |  |
| **35** | *asnA* | -1.58 |  |  |  |
| **36** | *feaR* | -1.58 |  |  |  |
| **37** | *acs* | -1.52 |  |  |  |
| **38** | *prpR* | -1.50 |  |  |  |
| **39** | *ytfQ* | -1.49 |  |  |  |
| **40** | *aldA* | -1.45 |  |  |  |

^a^Fold changes for each mRNA are in log2 scale in the wild type (WT) versus *sdsR* promoter mutant (*sdsRP^m^*) comparison.

**Table S5. RNA-seq analysis of mRNAs upregulated and downregulated over two-fold in *sdsR* promoter mutant cells at stationary phase (8 h)**

| **Upregulated** | | | **Downregulated** | | |
| --- | --- | --- | --- | --- | --- |
| **No.** | **Gene name** | **Fold change**  **(WT vs *sdsRP^m^*)^a^** | **No.** | **Gene name** | **Fold change**  **(WT vs *sdsRP^m^*)^a^** |
| **1** | *putA* | 6.07 | **1** | *insA* | -4.37 |
| **2** | *ryeA* | 3.09 | **2** | *ppdD* | -2.48 |
| **3** | *wcaF* | 3.01 | **3** | *yigG* | -2.48 |
| **4** | *rzpQ* | 2.84 | **4** | *ykgH* | -1.86 |
| **5** | *cysI* | 2.18 | **5** | *sfmH* | -1.74 |
| **6** | *cysH* | 2.05 | **6** | *agrB* | -1.66 |
| **7** | *cysA* | 2.00 | **7** | *leuL* | -1.60 |
| **8** | *cysW* | 1.98 | **8** | *astE* | -1.28 |
| **9** | *tfaP* | 1.91 | **9** | *astB* | -1.27 |
| **10** | *cysJ* | 1.90 | **10** | *yjjZ* | -1.25 |
| **11** | *cysU* | 1.87 | **11** | *astD* | -1.24 |
| **12** | *putP* | 1.77 | **12** | *yffN* | -1.22 |
| **13** | *cysC* | 1.62 | **13** | *astC* | -1.14 |
| **14** | *yeiL* | 1.60 | **14** | *yjhV* | -1.13 |
| **15** | *insA* | 1.57 | **15** | *astA* | -1.09 |
| **16** | *yeeE* | 1.54 | **16** | *puuA* | -1.08 |
| **17** | *cysN* | 1.53 | **17** | *yahL* | -1.07 |
| **18** | *cysD* | 1.52 | **18** | *mhpC* | -1.06 |
| **19** | *yeeD* | 1.47 | **19** | *azuC* | -1.05 |
| **20** | *stfP* | 1.43 | **20** | *puuD* | -1.04 |
| **21** | *cysP* | 1.35 | **21** | *ybhI* | -1.02 |
| **22** | *yciW* | 1.30 |  |  |  |
| **23** | *iraD* | 1.23 |  |  |  |
| **24** | *ydjN* | 1.20 |  |  |  |
| **25** | *yoeH* | 1.18 |  |  |  |
| **26** | *aphA* | 1.16 |  |  |  |
| **27** | *tolC* | 1.09 |  |  |  |
| **28** | *pyrB* | 1.09 |  |  |  |
| **29** | *cysK* | 1.08 |  |  |  |
| **30** | *cysM* | 1.08 |  |  |  |
| **31** | *ydfV* | 1.07 |  |  |  |
| **32** | *pyrI* | 1.06 |  |  |  |
| **33** | *borD* | 1.02 |  |  |  |

^a^Fold changes for each mRNA are in log2 scale in the wild type (WT) versus *sdsR* promoter mutant (*sdsRP^m^*) comparison.

Table S6. Validation of mRNAs identified, with more than 2-fold different expression, from RNA-seq data by qRT-PCR

| **Gene** | **Fold change**  **in RNA-seq^a^** | **Fold change**  **in qRT-PCR^a,b^** | **Description** |
| --- | --- | --- | --- |
| **WT vs *ryeAP^m^* (3 h)** | | | |
| *asnA* | -1.62 | -1.73 | Asparagine synthetase A |
| **WT vs *ryeAP^m^* (8 h)** | | | |
| *aphA* | -1.09 | -0.68 | Acid phosphatase/phosphotransferase |
| **WT vs *sdsRP^m^* (3 h)** | | | |
| *stpA* | 1.39 | 0.62 | H-NS-like DNA-binding transcriptional repressor with RNA chaperone activity |
| *ytfE* | 6.13 | 3.48 | Iron-sulfur cluster repair protein YtfE |
| *asnA* | -1.58 | -1.14 | Asparagine synthetase A |
| *ynfF* | -4.06 | -2.91 | Putative selenate reductase YnfF |
| \| **WT vs *sdsRP^m^* (8 h)** \| \| --- \| | | | |
| *aphA* | 1.16 | 0.62 | Acid phosphatase/phosphotransferase |
| *tolC* | 1.09 | 0.82 | Outer membrane channel TolC |
| *putA* | 6.07 | 4.66 | Fused DNA-binding transcriptional repressor |

^a^Fold changes for each mRNA are in log2 scale in the wild type (WT) versus *ryeA* or *sdsR* promoter mutant (*ryeAP*^m^ or *sdsRP^m^*) comparison.

^b^For all the relative expression data, P ≤ 0.05, by Student’s t-test.

Table S7. RNA-seq data of genes known as targets for SdsR

| Gene | Fold change^a^ | | | |
| --- | --- | --- | --- | --- |
|  | WT vs *ryeAP^m^* | | WT vs *sdsRP^m^* | |
|  | 3 h^b^ | 8 h^b^ | 3 h^b^ | 8 h^b^ |
| *yhcB* | -0.28 | -0.33 | 0.76 | 0.25 |
| *tolC* | -0.13 | -0.60 | -0.45 | 1.10 |
| *mutS* | 0.01 | -0.18 | -1.20 | 0.06 |

^a^Fold changes for each mRNA are in log2 scale in the wild type (WT) versus *ryeA* or *sdsR* promoter mutant (*ryeAP*^m^ or *sdsRP^m^*) comparison.

^b^Cells in exponential (3 h) or stationary phase (8 h).

**Supplementary figure legends**

**Supplementary Figure 1. Reciprocal expression and their regulation between RyeA and SdsR (extended)*.*** The Northern membrane of Fig. 3A probed by the anti-RyeA probe was stripped and rehybridized with the anti-SdsR probe. SdsR(p), the processed form of SdsR.

**Supplementary Figure 2. Mutual degradation of RyeA and SdsR.** (A) A schematic of the workflow used to ectopically co-express RyeA and SdsR. RyeA or SdsR was first induced by arabinose for 5 min and 25 min. Then the 5 min- or 25 min-induced cells were treated with IPTG to induce expression of the other RNA and collected for RNA analysis in time intervals. (B) RyeA was induced first by 0.2 % arabinose, then SdsR by 1 mM IPTG in *sdsR/ryeA* deletion mutant cells (MG1655*ΔsdsR/ryeA*) containing plasmids pSdsR and pRyeA-ara. SdsR and RyeA were analyzed by Northern blotting. The Northern membrane was probed by the anti-RyeA probe, stripped and reprobed with the anti-SdsR probe. (C) SdsR was induced first by 0.2 % arabinose, then RyeA by 1 mM IPTG in *sdsR/ryeA* deletion mutant cells (MG1655*ΔsdsR/ryeA*) containing plasmids pRyeA and pSdsR-ara. SdsR and RyeA were analyzed by Northern blotting as in (B). pRyeA and pSdsR, IPTG-induced RyeA and SdsR expression plasmids derived from pBR322, respectively. pRyeA-ara and pSdsR-ara, arabinose-induced RyeA and SdsR expression plasmids from pACYC184.

**Supplementary Figure 3. Ontology diagrams of RNA-seq analysis.** Genes with more than a 2-fold change in expression in *sdsR* promoter mutant cells relative to WT cells in the exponential phase (A) and stationary phase (B) were categorized according biological process and cellular components. E, exponential phase (3 h); S, stationary phase (8 h).

**Supplementary Figure 4. Reexamination of promoters for *pphA* transcription. (A)** The region -379 to +222 containing the previously reported *pphA* promoter as well as the *sdsR* promoter was cloned into pKK232-8 to generate the *sdsR*(-379/+131)-CAT plasmid. The nucleotide sequences for the cloned region are shown. (B) Total RNAs were prepared from MG1655Δ*ryeA/sdsR* cells carrying the *sdsR*(-379/+131)-CAT plasmid grown for 2 h, 6 h, and 10 h. Primer extension analysis was performed as described in Fig. 1B except that primer SdsR+59R, which binds to the +58 to +75 of SdsR, was used. Products from 6 h and 10 h were loaded after diluting 1/20 and 1/2, respectively.

**Supplementary Figure 5. Reduction of *pphA* expression and delay of its expression time by *ryeA* transcription during growth.** (A) DJ480Δ*ryeA/sdsR* cells carrying the *sdsR*(-79/+222)-*lacZ* or *sdsR*(-79/+222*ryeAP^m^*)-*lacZ* transcriptional fusion were analyzed for LacZ activities during growth at at 37°C. *ryeAP^m^*, *ryeA* -10 promoter mutation (mean ± SD; n = 3). Optical density (OD) at 600 nm was measured for cell growth.

**Supplementary Figure 6. Effects of novobiocin on interference of *sdsR* transcription by *ryeA* transcription.** (A) Concentration of novobiocin required for the 50% inhibition of growth (IC_50_) of DJ480Δ*ryeA/sdsR* cells carrying the *sdsR*(-379/+222)-*lacZ* fusion was determined as 100 μg/ml. (B) DJ480Δ*ryeA/sdsR* cells carrying the *sdsR*(-379/+222)-*lacZ* or *sdsR*(379/+222*ryeAP^m^*)-*lacZ* fusion were grown for 2 h (E) and 10 h (S), and analyzed for LacZ activities at 100 μg/ml of novobiocin. *ryeAP^m^*, *ryeA* -10 promoter mutation. E, exponential phase; S, stationary phase.

**Supplementary Figure 7. Growth phase-dependence of *ryeA* and *sdsR* promoter activities.** (A) DJ480 cells carrying the *ryeA* promoter-*lacZ* or *sdsR* promoter-*lacZ* fusion construct were analyzed for LacZ activities during growth at 37°C. Cell growth was monitored by measuring optical density (OD) at 600 nm. (B). Ratios between LacZ activities from the *ryeA* and *sdsR* promoters were plotted during growth.

**Figure S1**

**
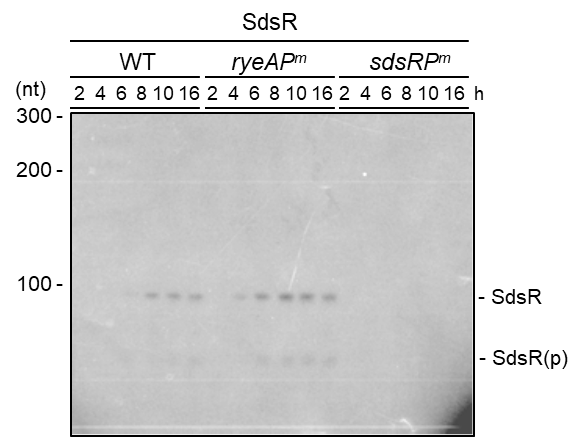
**

**Figure S2**

**
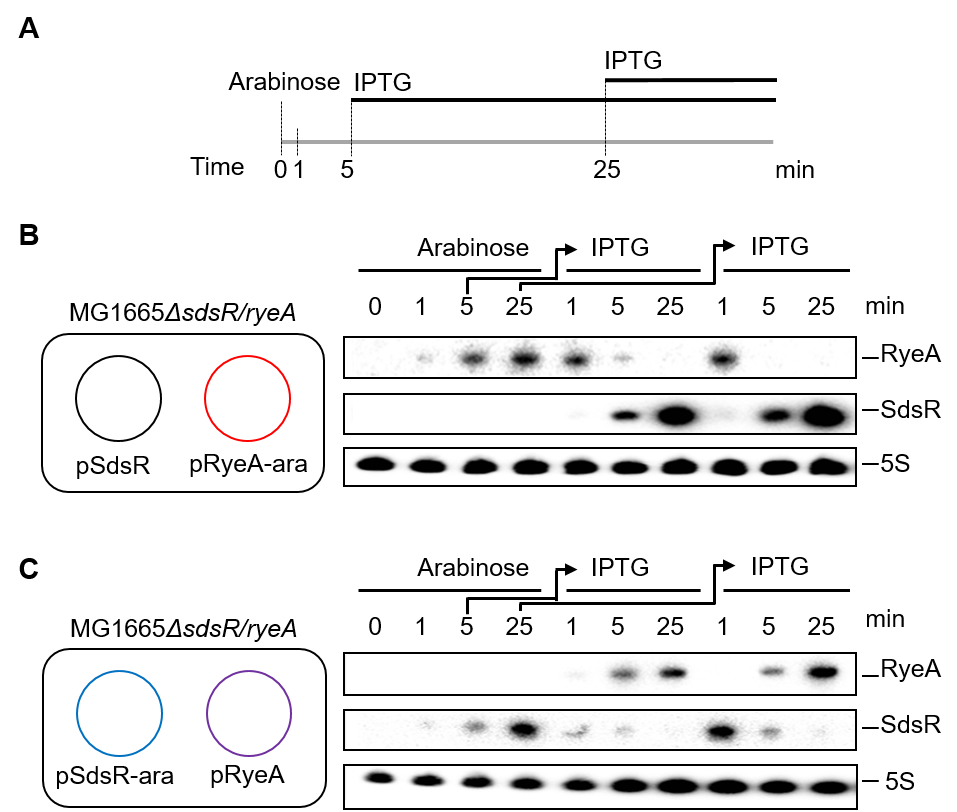
**

**Figure S3
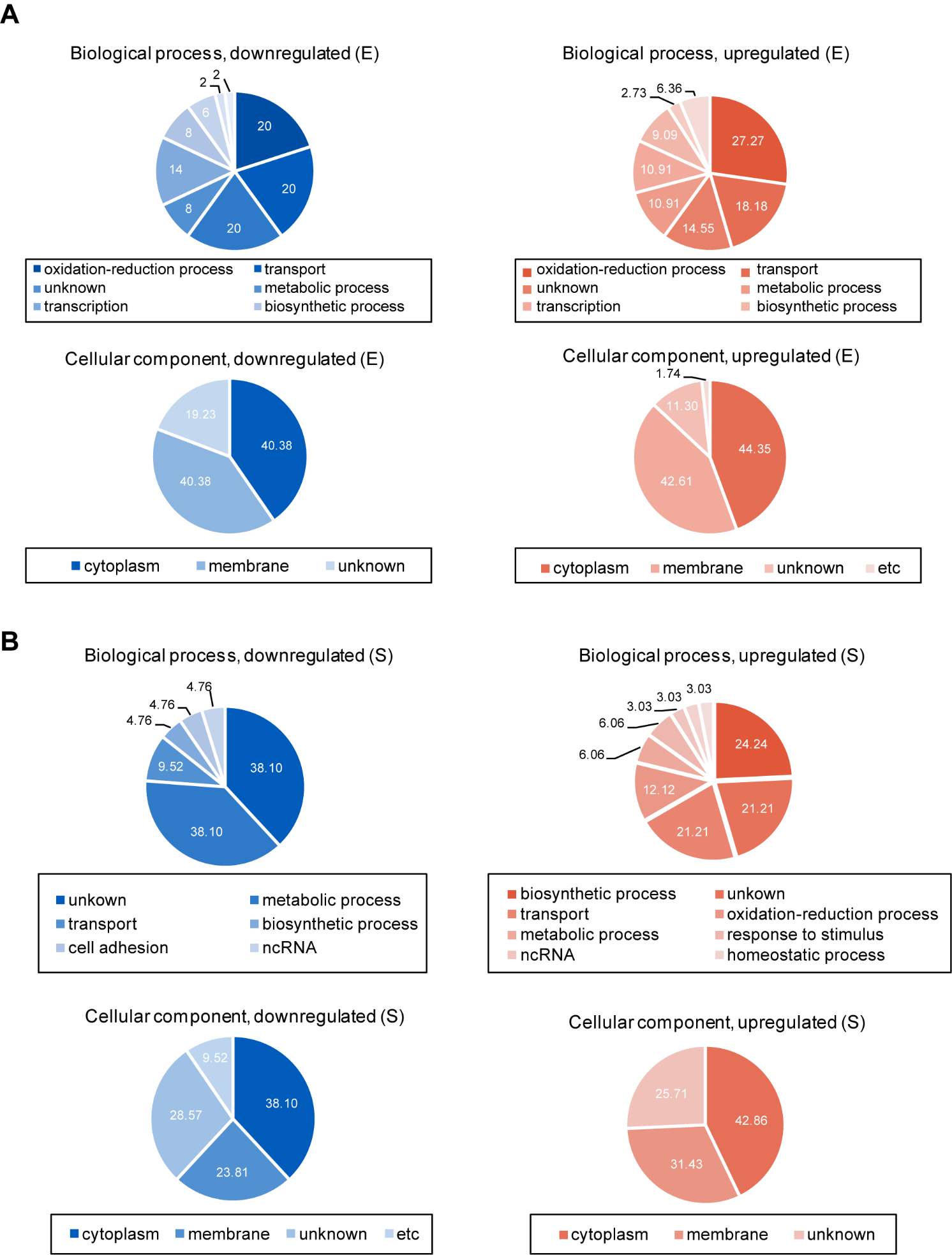
**

**Figure S4**

**
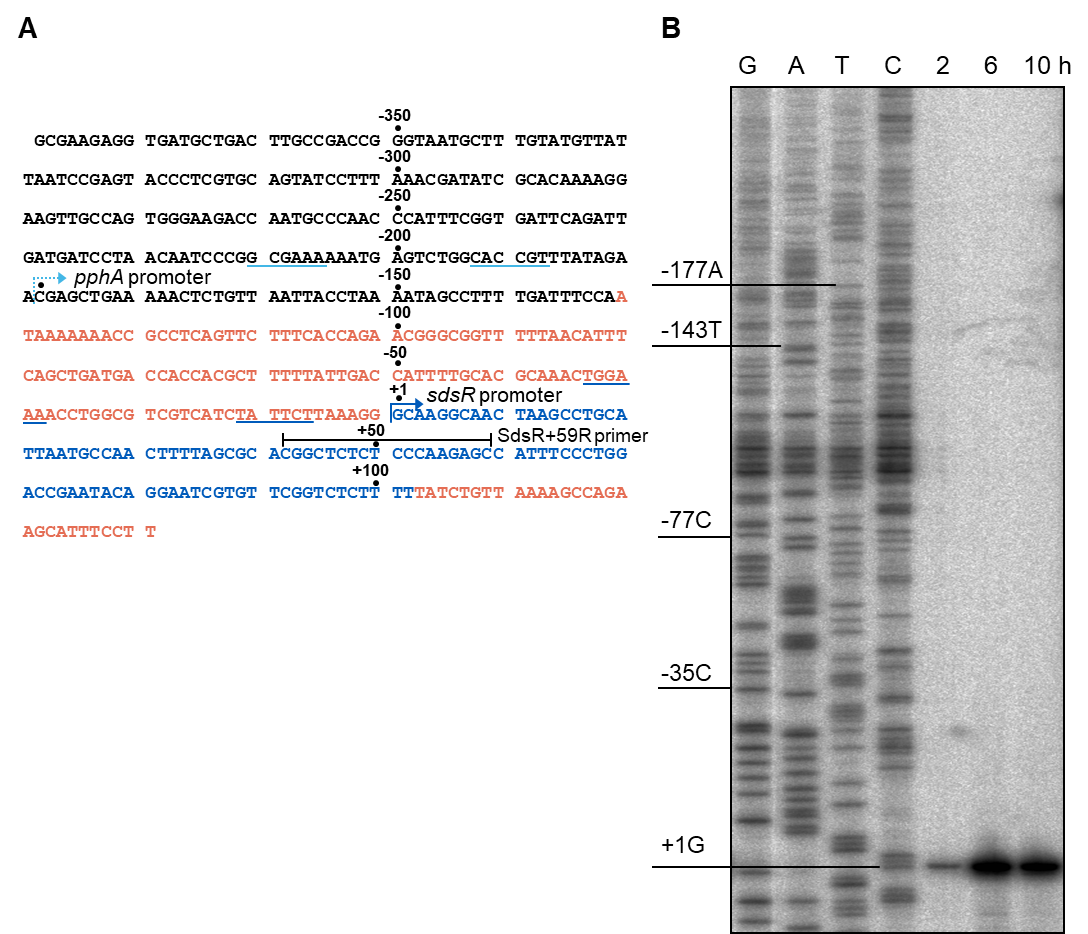
**

**Figure S5**


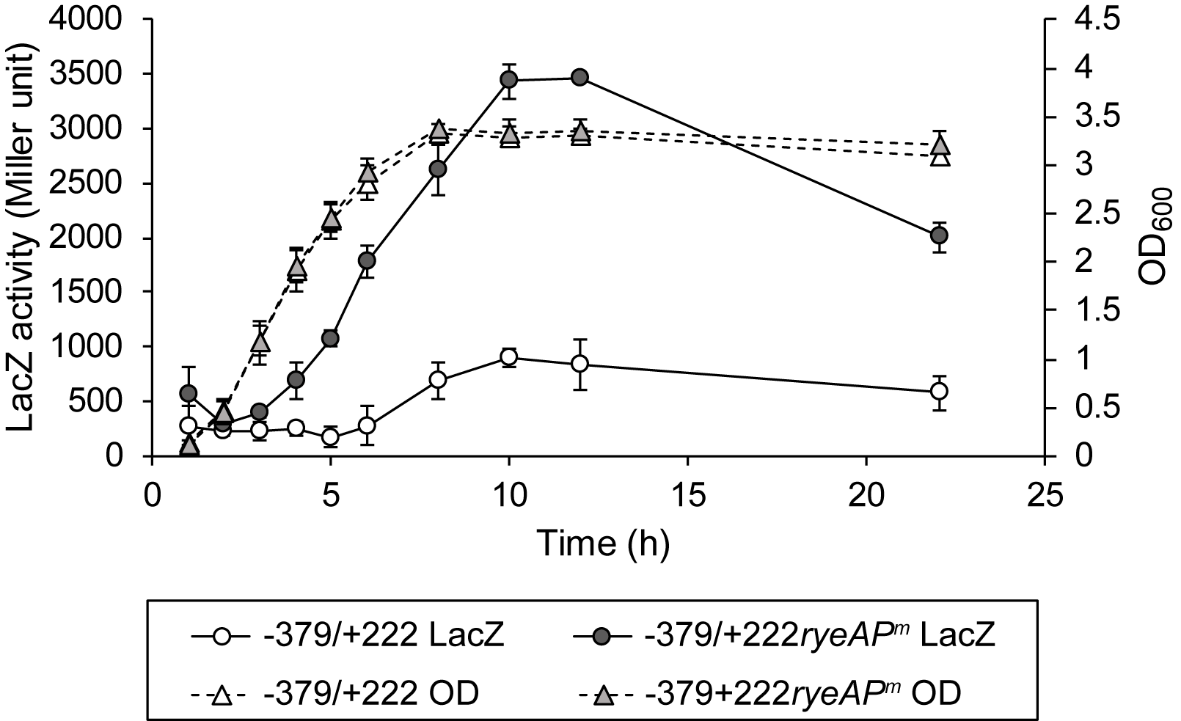


**Figure S6**

**
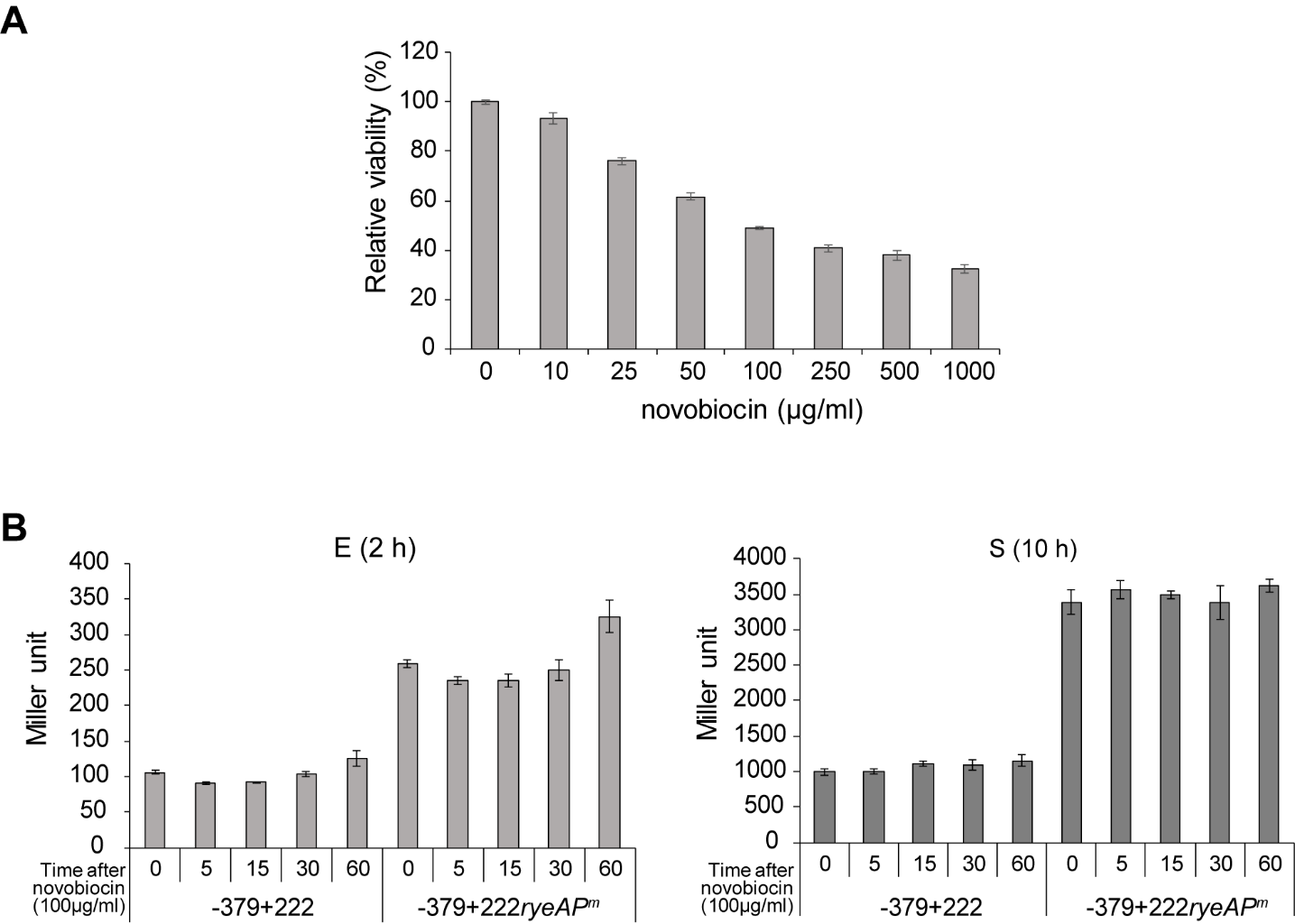
**

**Figure S7**

**
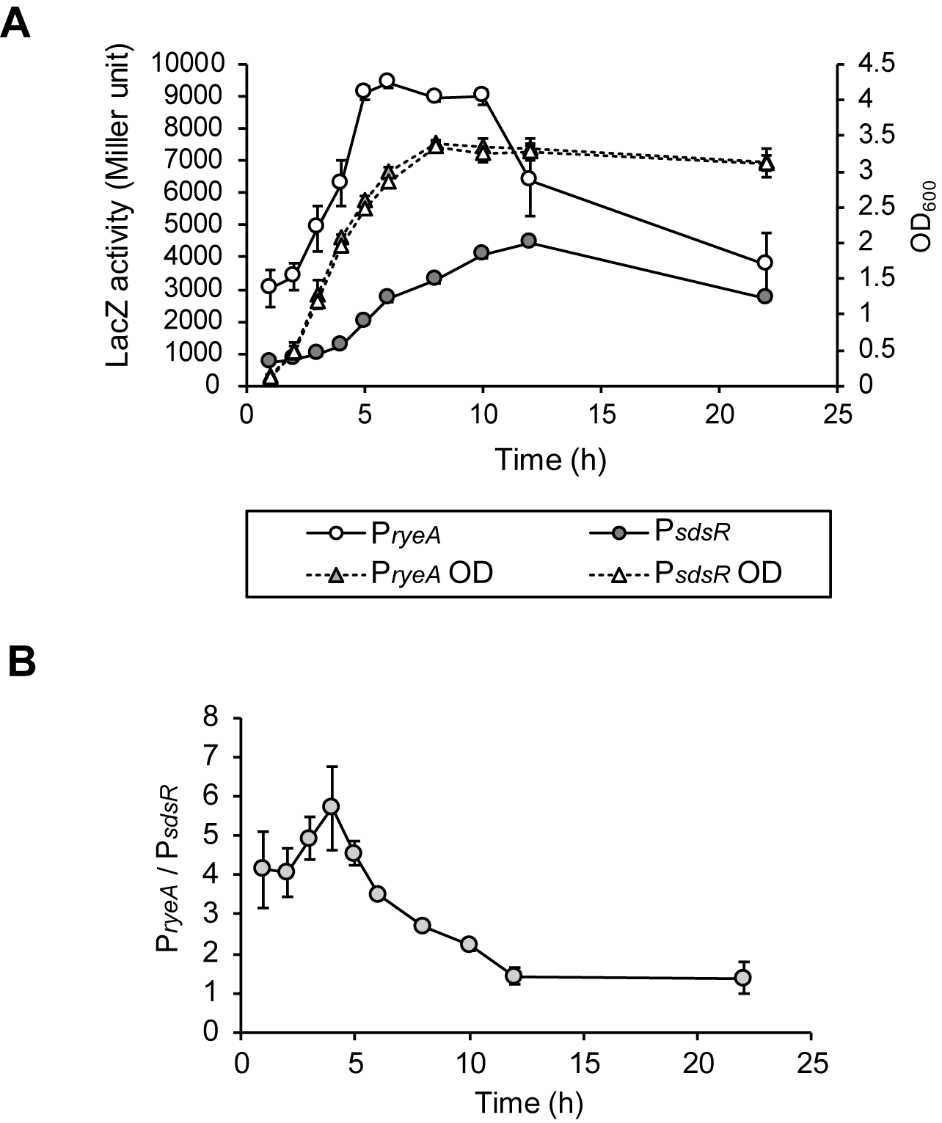
**
